# Improved photovoltaic performance and robustness of all-polymer solar cells enabled by a polyfullerene guest acceptor

**DOI:** 10.1038/s41467-023-37738-9

**Published:** 2023-04-22

**Authors:** Han Yu, Yan Wang, Xinhui Zou, Junli Yin, Xiaoyu Shi, Yuhao Li, Heng Zhao, Lingyuan Wang, Ho Ming Ng, Bosen Zou, Xinhui Lu, Kam Sing Wong, Wei Ma, Zonglong Zhu, He Yan, Shangshang Chen

**Affiliations:** 1 State Key Laboratory of Coordination Chemistry, MOE Key Laboratory of High-Performance Polymer Materials & Technology, School of Chemistry and Chemical Engineering, Nanjing University, 210023 Nanjing, Jiangsu China; 2 Department of Chemistry, Guangdong-Hong Kong-Macao Joint Laboratory of Optoelectronic and Magnetic Functional Materials, Energy Institute and Hong Kong Branch of Chinese National Engineering Research Center for Tissue Restoration & Reconstruction, Hong Kong University of Science and Technology, Clear Water Bay, 999077 Kowloon, Hong Kong China; 3 Department of Chemistry and Hong Kong Institute for Clean Energy, City University of Hong Kong, 999077 Kowloon, Hong Kong China; 4 Department of Physics, Hong Kong University of Science and Technology, Clear Water Bay, 999077 Kowloon, Hong Kong China; 5 Department of Physics, Chinese University of Hong Kong, 999077 New Territories, Hong Kong China; 6 State Key Laboratory for Mechanical Behavior of Materials, Xi’an Jiaotong University, 710049 Xi’an, China

**Keywords:** Electronic devices, Solar cells, Conjugated polymers, Devices for energy harvesting

## Abstract

Fullerene acceptors typically possess excellent electron-transporting properties and can work as guest components in ternary organic solar cells to enhance the charge extraction and efficiencies. However, conventional fullerene small molecules typically suffer from undesirable segregation and dimerization, thus limiting their applications in organic solar cells. Herein we report the use of a poly(fullerene-*alt*-xylene) acceptor (PFBO-C12) as guest component enables a significant efficiency increase from 16.9% for binary cells to 18.0% for ternary all-polymer solar cells. Ultrafast optic and optoelectronic studies unveil that PFBO-C12 can facilitate hole transfer and suppress charge recombination. Morphological investigations show that the ternary blends maintain a favorable morphology with high crystallinity and smaller domain size. Meanwhile, the introduction of PFBO-C12 reduces voltage loss and enables all-polymer solar cells with excellent light stability and mechanical durability in flexible devices. This work demonstrates that introducing polyfullerenes as guest components is an effective approach to achieving highly efficient ternary all-polymer solar cells with good stability and mechanical robustness.

## Abstract (web summary)

Fullerene acceptors typically suffer from undesirable segregation and dimerization. Here, the authors report a poly(fullerene-alt-xylene) acceptor as guest component to facilitate charge transfer and suppress charge recombination, achieving efficiency of 18% for ternary all-polymer solar cells.

## Introduction

Organic solar cells (OSCs) have attracted considerable attention from both academia and industry due to their portability, transparency, flexibility, and facile fabrication^1–4^. Owing to the extensive research efforts devoted to material development and device optimization^5–7^, the power conversion efficiencies (PCEs) of OSCs based on small-molecular acceptors (SMAs) have exceeded 19% recently^8–11^, demonstrating their great potential as one of the most promising emerging solar technologies. Despite that, the device stability issue still remains a critical factor that limits the commercialization of SMA OSCs. To this end, all-polymer solar cells (all-PSCs), which employ both polymeric donors and acceptors as light absorbers (Supplementary Fig. 1), have been attracting the OSC community’s attention due to their additional advantages of robust mechanical toughness, and excellent light/thermal stability for commercial applications^12–16^. With the development of Y-series SMAs^17–20^ and their derived polymerized-SMAs (Supplementary Fig. 2)^21–28^, the all-PSCs based on Y-series polymer acceptors have realized decent efficiencies of over 17%^29–34^.

To further improve the PCEs of all-PSCs, constructing a ternary cell by introducing a guest component into binary host systems has been proven an effective and facile approach^29,35–37^. Regarding guest components, currently, one type of the most widely used guest materials of ternary OSCs are fullerene molecules (e.g., Phenyl-C_61_-butyric acid methyl ester (PCBM))^38,39^, which exhibit excellent electron-withdrawing properties and can enhance carrier transporting of OSCs^40^. Thus far, there have been a few reports of using PCBM as the guest component for ternary all-PSCs to improve device efficiencies^41,42^. Despite the good electron-accepting property of PCBM, its intrinsic instability remains a non-ignorable factor impairing device stability^43,44^. For instance, it has been found that PCBM molecules tend to dimerize under light illuminance and the dimerization significantly reduces the charge-transporting capacity of PCBM^45,46^. Meanwhile, the PCBM molecules are prone to aggregate into large clusters that decrease film mechanical robustness. Unfortunately, few alternatives have been reported that exhibit both comparable electron-transporting properties and good stability to replace PCBM or its derivatives.

In this work, we propose a new strategy for developing highly efficient and stable all-PSCs by replacing conventional PCBM molecules with polyfullerene materials. A poly(fullerene-*alt*-xylene) acceptor, named PFBO-C12, was synthesized and introduced as the guest component in ternary all-PSCs. Ultrafast optic and optoelectronic characterizations reveal that PFBO-C12 can promote the hole transfer process and suppress charge recombination. In addition, morphological characterizations show that the ternary blend maintained high crystallinity for fast carrier transport and more suitable phase separation with a smaller domain size for charge dissociation. As a result, the poly(fullerene-*alt*-xylene)-based ternary all-PSCs achieve an impressive PCE of 18.0%, and the device stability of ternary all-PSC is significantly improved compared to the PCBM counterpart. Meanwhile, PFBO-C12-based flexible all-PSCs are found to exhibit excellent bending durability in flexible devices, which maintained 90% of initial efficiency after 1000-time bending cycles. Overall, our work demonstrates that PFBO-C12 can work as an effective guest component to achieve high-performance all-PSCs with good efficiencies, stability, and mechanical toughness.

## Results

### Materials and optoelectronic characterization

**Fig. 1:**
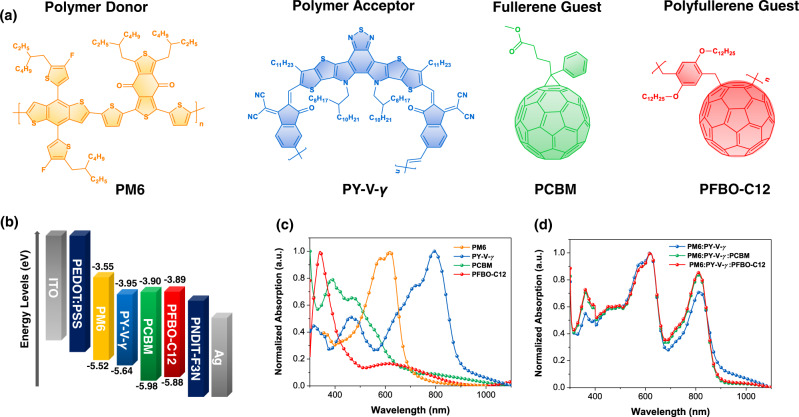
Materials and optoelectronic characterization. **a** Chemical structures of PM6 (polymer donor), PY-V-*γ* (polymer acceptor), PCBM (fullerene guest), and PFBO-C12 (polyfullerene guest). **b** Architecture and energy alignment of the materials in all-PSCs. Normalized UV–Vis absorption spectra of **c** neat and **d** blend films. Source data are provided as a Source Data file.

The chemical structures of PM6, PY-V-*γ*, PCBM, and PFBO-C12 are shown in Fig. 1a. The polymer acceptor PY-V-*γ* was synthesized according to our previous works^30,47^. PCBM is chosen as another guest component for comparison. The synthesis details of the PFBO-C12 poly(fullerene-*alt*-xylene) are provided in Supporting Information^48,49^. The molecular weight of PFBO-C12 was measured to be 8802 g mol^−1^ (*M*_n_) and 18,947 g mol^−1^ (*M*_w_) by high-temperature gel permeation chromatography (HT-GPC), it should be noted that the measurement inaccuracy still remains due to the structure difference between polyfullerene and polystyrene standard. Cyclic voltammetry (CV, Fig. 1b, Supplementary Fig. 3) was used to estimate the energy levels of the materials in this work. It shows that PFBO-C12 exhibits comparable energy levels to PCBM. The ultraviolet–visible (UV–Vis) absorption spectra of the four materials were depicted in Fig. 1c. The maximum absorption peak (*λ*_max, film_) of PFBO-C12 is located at 340 nm, together with a broader shoulder absorption peak at 650 nm. Similar to PCBM, PFBO-C12 forms a complementary absorption to PM6 and PY-V-*γ*. The absorption spectra of PM6:guest acceptor (10% weight ratio) films exhibit the 0–0 peak enhancement from PCBM to PFBO-C12 (Supplementary Fig. 4a), which suggests a proper addition of PFBO-C12 can further promote the aggregation of PM6. Regarding the majority polymer acceptor in films, introducing the fullerene/polyfullerene guest acceptors was found to strengthen the intensity of PY-V-*γ* at *λ*_max, film_ (~830 nm), implying their comparable miscibility and co-crystallization tendency in films (Supplementary Fig. 4c). As a result, both ternary systems display total absorption enhancement from 300 to 800 nm relative to the binary combination (Fig. 1d). The PFBO-C12-based blend shows even stronger intensity from 300 to 500 nm, which is beneficial for photon harvesting and photocurrent generation.

### Photovoltaic device performance

**Fig. 2:**
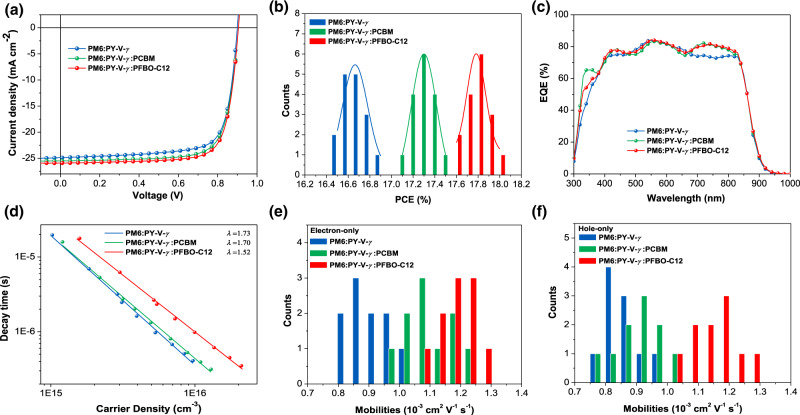
Photovoltaic performance, charge recombination, and carrier mobility. **a** Current density–voltage (*J–V*) characteristics, **b** power conversion efficiency (PCE) statistical histograms, **c** external quantum efficiency (EQE) spectra, and **d** transient photovoltage (TPV) decay time as a function of carrier density of three groups of all-PSCs. Statistical histograms of **e** electron and **f** hole mobility of the devices based on three types of blends. Source data are provided as a Source Data file.

To investigate the effects of PFBO-C12 on photovoltaic performance, all-PSCs were fabricated using a conventional device architecture of glass/ITO/PEDOT:PSS/active layer/PNDIT-F3N/Ag by a sequential deposition method (see details in the “Methods” section). After blending with PM6, the current-density-versus-voltage (*J*–*V*) curves of the three optimized devices were plotted in Fig. 2a, and the corresponding photovoltaic parameters of three groups of all-PSCs are summarized in Table 1. By constructing a pseudo-bilayer architecture^50–52^, the PCEs of both ternary cells increase compared to the binary system (Fig. 2b). PM6:PY-V-*γ*:PFBO-C12 based all-PSCs achieved the highest PCE of 18.0% with a high *V*_OC_ (or small voltage loss), enhanced *J*_SC_ and FF relative to the other two material combinations, which is also the record PCE for all-PSCs reported before the submission of this work (Supplementary Table 1). Next, we carried out external quantum efficiency (EQE) measurements to study the changes in *J*_SC_s of the optimized devices (Fig. 2c). After introducing the PCBM/PFBO-C12 guest components, the EQE response enhances from 670 to 830 nm, which is attributed to the stronger conjugation absorption in this range of the ternary blends. Meanwhile, the EQE response of the PFBO-C12-based all-PSCs slightly increases from 460 to 680 nm relative to the other two systems, leading to the highest *J*_cal_ of 25.1 mA cm^−2^. Overall, the integrated *J*_SC_s calculated from the EQE spectra match well with the values attained from the *J–V* curves (Table 1) and are consistent with the trend observed in UV–Vis absorption spectra.

**Table 1:** Photovoltaic parameters of the three groups of all-PSCs under the AM 1.5 G illumination intensity of 100 mW cm^−2^

Materials	*V*_OC_ [V]	*J*_SC_ [mA cm^−2^]	*J*_cal_ [mA cm^−2^]	FF [%]	PCE^a^ [%]
PM6:PY-V-*γ*	0.900 (0.897 ± 0.003)	24.9 (24.7 ± 0.2)	24.3	75.2 (74.9 ± 0.3)	16.9 (16.6 ± 0.3)
PM6:PY-V-*γ*: PCBM	0.904 (0.902 ± 0.002)	25.5 (25.3 ± 0.2)	24.9	76.1 (75.7 ± 0.4)	17.5 (17.3 ± 0.2)
PM6:PY-V-*γ*: PFBO-C12	0.905 (0.903 ± 0.002)	25.8 (25.6 ± 0.2)	25.1	77.0 (76.8 ± 0.2)	18.0 (17.8 ± 0.2)

^a^Average values from 16 individual devices with the average values shown in parentheses.

### Charge dissociation, recombination, and transport

Time-resolved photoluminescence (TR-PL) characterizations were performed to study their exciton dissociation behaviors (Supplementary Fig. 5a). The pristine PY-V-*γ* exhibited a PL decay lifetime of 0.38 ns and decreased to 0.20 ns after mixing with PM6 as the binary blend. As for ternary blends, the PL quenching process becomes even faster as the guest components were introduced, indicating both PCBM (0.19 ns) and PFBO-C12 (0.16 ns) help exciton dissociation. The trend agrees well with exciton dissociation probability (*P*, Supplementary Fig. 5b) measurement results that the PFBO-C12-based ternary devices showed the highest *P* among the three types of all-PSCs^53^.

Transient photocurrent (TPC) and transient photovoltage (TPV) measurements were conducted to study the charge extraction and recombination processes. In addition to the TPV spectra obtained under one sun illumination (Supplementary Fig. 6a, b)^54^, we also performed TPV characterization under different *V*_OC_ levels via tuning the intensity of background light illumination to better discern their differences in charge recombination (Supplementary Fig. 6c, d). The photogenerated carrier density is calculated via TPC measurements under corresponding conditions. The TPV decay time *τ* under different *V*_OC_ follows the power law with the calculated carrier density *n* (equation (1): *τ* = *τ*_0_(*n*/*n*_0_)^−*λ*^, and (*λ* + 1) represents the charge carrier recombination order)^55,56^. The carrier dependence of the TPV decay time of the three systems is shown in Fig. 2d. The fitted exponent *λ* are 1.73, 1.70, and 1.52 for the binary, PCBM and PFBO-C12-based all-PSCs, respectively. The binary system is found to have a higher recombination order (*λ* + 1), which indicates its more severe trap-assisted recombination. The less dependence on the decay time of the ternary systems suggests dominant bimolecular recombination for charge carriers and the trap-assisted recombination is greatly reduced in the PFBO-C12 ternary system. These results are further supported by light-intensity-dependent *J*_SC_ and *V*_OC_ characterizations of the three types of all-PSCs (Supplementary Fig. 6e, f), which show that PM6:PY-V-*γ*:PFBO-C12-based device exhibited suppressed bimolecular and trap-assisted charge recombination respectively, thus contributing to a higher FF.

The hole (*μ*_h_) and electron (*μ*_e_) mobilities of the three all-polymer blends were characterized via the space-charge-limit current (SCLC) method (Supplementary Fig. 7)^57^. As shown in Fig. 2e, f and Supplementary Table 2, the *μ*_h_/*μ*_e_ of PM6:PY-V-*γ* were measured to be 8.6 × 10^–4^/9.0 × 10^–4^ cm^2^ V^–1^ s^–1^, which increased to 9.1 × 10^–4^/1.1 × 10^–3^ cm^2^ V^–1^ s^–1^ for PM6:PY-V-*γ*:PCBM blend. In comparison, PM6:PY-V-*γ*:PFBO-C12 exhibits the highest *μ*_e_ of 1.2 × 10^–3^ cm^2^ V^–1^ s^–1^ and a superior *μ*_h_ of 1.1 × 10^–3^ cm^2^ V^–1^ s^–1^ among three groups of devices. Moreover, PFBO-C12-based blends show a more balanced *μ*_h_/*μ*_e_ value, which reduces charge accumulation and enables efficient charge extraction for high FFs^58^.

### Morphology characterization and energy loss analysis

**Fig. 3:**
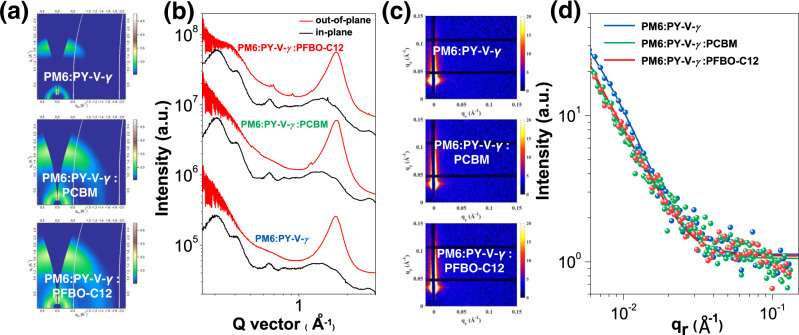
Morphology characterization. **a** 2D GIWAXS patterns of the blend films and **b** the corresponding 1D GIWAXS line-cuts of the in-plane (black line) and out-of-plane (red line) directions. **c** 2D GISAXS patterns of the blend films and **d** the corresponding GISAXS intensity profiles and best fittings along the in-plane directions. Source data are provided as a Source Data file.

Grazing incidence wide-angle X-ray scattering (GIWAXS) characterizations can disclose the morphology characteristics of the films, and enable us to study the effects of the fullerene/poly(fullerene-*alt*-xylene) guests on film crystallinity and orientations^59^. As shown in Fig. 3a, b and Supplementary Table 3, all three blends exhibit similar π–π (010) stacking peaks located at ≈1.7 Å^–1^ in the out-of-plane direction, together with comparable coherent lengths (CLs) of 27 nm (dominated by PM6, Supplementary Fig. 8). In the in-plane direction, they also exhibit strong lamellar (100) peaks at *q*_*z*_ ≈ 0.30 Å^−1^, implying the face-on orientation is also dominant in the blends that facilitate charge transport in the vertical direction. Meanwhile, both (100) and (200) peaks of the PFBO-C12-based blend show longer CLs relative to the other two combinations, indicative of a higher crystallinity for faster charge transport enabled by PFBO-C12. Grazing-incidence small-angle X-ray scattering (GISAXS) experiments were also involved to evaluate the impacts of guest components on the phase segregation of the blends^60^. Figure 3c, d shows the GISAXS results of the three all-PSCs, where the crystalline domain size (2*R*_g_) of the ternary blends became smaller as the fullerene/poly(fullerene-*alt*-xylene) guest added, beneficial for charge dissociation as discussed above. Besides, the intermixing domain spacings (*χ*) decreased from 43.0 for the binary film, to 38.1 and 35.9 nm for PCBM- and PFBO-C12-based ternary blends. The higher domain purity of PFBO-C12-based blends will enable more effective charge extraction and suppress the charge recombination, which could be the major reason for their superior FFs and PCEs.

Furthermore, we looked into the energy loss (*E*_loss_) among the three all-PSCs by carrying out in-depth photophysical experiments (Supplementary Fig. 9, Supplementary Table 4)^61^. A small energy offset between donors and acceptors can effectively suppress the charge-transfer (CT) state recombination through the hybridization of the local excited (LE) state and the CT state, resulting in reduced non-radiative recombination loss^62^. Due to the higher occupied orbitals of the guest components, the energy level offsets decrease between PM6 and the acceptors when the guest component is mixed with the PY-V-*γ*. Therefore, both ternary devices exhibit smaller Δ*E*_3_ relative to the binary devices. The final *E*_loss_s were determined to be 0.528, 0.521, and 0.514 eV for PM6:PY-V-*γ*, PM6:PY-V-*γ*:PCBM, and PM6:PY-V-*γ*:PFBO-C12 cells, respectively. The smallest *E*_loss_ of the PFBO-C12-based device should mainly result from the higher degree of conformational order of the polymer acceptor, with denser packing, fewer vibration states, and suppressed non-radiative recombination as above.

### Exciton and charge dynamics study

**Fig. 4:**
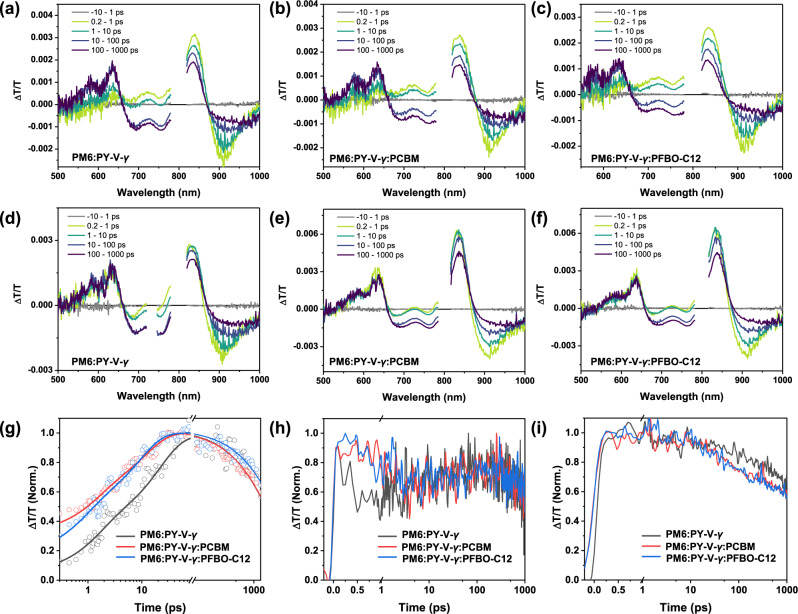
Exciton and charge dynamics. Transient absorption spectra (TAS) of **a** PM6:PY-V-*γ*, **b** PM6:PY-V-*γ*:PCBM, and **c** PM6:PY-V-*γ*:PFBO-C12 blend films excited at 800 nm. TAS of **d** PM6:PY-V-*γ*, **e** PM6:PY-V-*γ*:PCBM, and **f** PM6:PY-V-*γ*:PFBO-C12 blend films excited at 370 nm. Kinetics of GSB of PM6 (630–640 nm) for PM6:PY-V-*γ*, PM6:PY-V-*γ*:PCBM and PM6:PY-V-*γ*:PFBO-C12 blends excited at **g** 800 nm and **h** 370 nm, respectively. **i** Kinetics of GSB of PY-V-*γ* (820–840 nm) for PM6:PY-V-*γ*, PM6:PY-V-*γ*:PCBM and PM6:PY-V-*γ*:PFBO-C12 blends excited at 370 nm. Source data are provided as a Source Data file.

Charge transfer processes in the blend films were investigated via transient absorption spectra (TAS) measurement. First, three blend films were excited at 800 nm, and the immediate rise of the ground-state bleach (GSB) signal (820–840 nm) corresponds to the absorption of PY-V-*γ* (Fig. 4a–c). The photoinduced absorption (PA) assigned to PY-V-*γ* at ~910 nm also emerges immediately after pump excitation. After 10 ps, a new PA band at ~960 nm emerges and starts to grow while the PA of PY-V-*γ* decays. Since PY-V-*γ* is selectively excited, the rise of PM6 GSB at ~640 nm can only result from the hole transfer from the acceptor to PM6, thus allowing us to probe the hole transfer process by monitoring the GSB of PM6. As shown in Fig. 4g, the hole transfer process in ternary blends is much faster than that in the binary. A bi-exponential model was introduced to fit the rise of donor GSB^63^. The rise lifetime *τ*_1_/*τ*_2_ are 1.48/21.37, 1.28/11.71, and 0.79/9.06 ps for the PM6:PY-V-*γ*, PM6:PY-V-*γ*:PCBM, and PM6:PY-V-*γ*:PFBO-C12, respectively (*τ*_1_ can be attributed to the exciton dissociation time at donor/acceptor interface, while *τ*_2_ represents the exciton diffusion time to the interface). These results indicate both PCBM and PFBO-C12 facilitate the hole transfer from PY-V-*γ* to PM6, and PFBO-C12 works more effectively on that. Besides, it is also noteworthy that the lifetime of the donor GSB signal of the PFBO-C12-based blend is slower (~3 ns), which is longer than the other two blends (~2 ns), implying a suppressed bimolecular recombination process in the polyfullerene system.

Subsequently, we preferentially excite the fullerene absorption region at 370 nm (Fig. 4d–f), and we find a higher intensity of TA response for the ternary blend, which is due to the stronger absorption in the UV region. As shown in Fig. 4h, the donor GSB emerges within our measurement time resolution (~150 fs) in all the blends, while the ternary blends show a faster rise of the acceptor GSB. We believe this is due to the Förster resonance energy transfer (FRET) from the fullerenes to PY-V-*γ*. A similar trend is also observed in the steady-state PL measurements (Supplementary Fig. 4d) that PY-V-*γ* shows PL enhancement when blended with fullerene components^64^. For the binary blend, there is only FRET from donor to acceptor. This also agrees with that the two ternary blends show stronger acceptor GSB signals than that of the binary blend. In addition, we also observe a slower decay of the acceptor GSB signal for the binary blend after 10 ps (Fig. 4i). This timescale corresponds to the rise of donor GSB after the fast electron transfer process from donor to acceptor. Since hole transfer leads to the significant decrease of acceptor GSB signal (as suggested by 800 nm excitation TA), this slower decay also suggests the slower hole transfer of the binary blend.

### Device stability and mechanical robustness

**Fig. 5:**
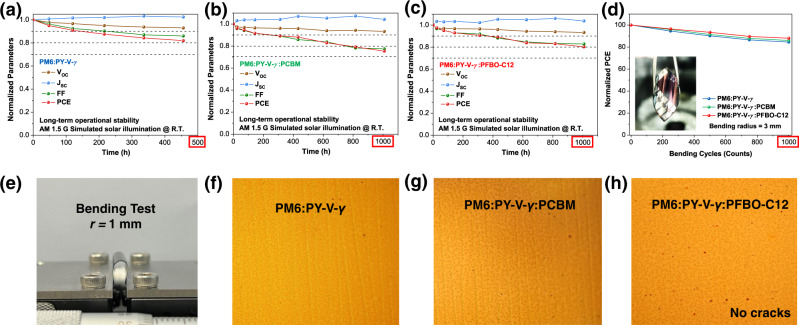
Light stability, flexible device and mechanical test. **a**–**c** Light-soaking stability test results of the encapsulated all-PSCs based on three blends under 1-sun illumination at room temperature. **d** Bending durability tests of the flexible all-PSCs as a function of bending cycles with a radius of 3 mm. **e**–**h** Photo images of flexible all-PSCs under the optical microscope after bending test at a bending radius of 1 mm. Source data are provided as a Source Data file.

We also look into the effects of fullerene guest components on device stabilities. Light-soaking tests were first conducted to evaluate the long-term operational stability of the three groups of all-PSCs. As shown in Fig. 5a–c, the PFBO-C12-based device showed the best light stability with a *T*_80_ of 1000 h under continuous 1-sun illumination, while both binary and PCBM-based ternary devices suffered from fast decay in FFs following with shorter *T*_80_s of 500 and 800 h, respectively. This result demonstrates the stabilization effect of PFBO-C12 in ternary cells. Subsequently, the flexible all-PSCs were fabricated with a structure of polyethylene naphthalate (PEN)/ITO/PEDOT:PSS/active layer/PNDIT-F3N/Ag to further investigate mechanical stability in flexible devices. The PM6:PY-V-*γ*:PFBO-C12-based device achieved the highest PCE of 14.6% among three flexible devices (Supplementary Fig. 10a and Supplementary Table 5), which is also one of the best efficiencies for flexible all-PSCs^32,65^. As shown in Fig. 5e–h, the PFBO-C12-based flexible devices display no obvious fracture at a bending radius (*r*) of 1 mm, relative to the flat device, demonstrating its intrinsic toughness after PFBO-C12 was introduced. We further tested the bending durability of the three groups of all-PSC as shown in Fig. 5d. PM6:PY-V-*γ*:PFBO-C12-based all-PSC maintained over 90% of the initial PCE after 1000 bending cycles, outperforming the other two types of devices. Such a good mechanical performance is mainly attributed to the elasticity of the PFBO-C12 polymer that enables the flexible cells more robust to mechanical bending.

To estimate the universality of the poly(fullerene-*alt*-xylene) guest, we choose another state-of-the-art all-polymer material combination of PM6:PYF-T-*o* as the host^22^, following the same procedure in device fabrication. It was found that both ternary devices showed enhanced FFs and PCEs, where the PM6:PYF-T-*o*:PFBO-C12-based devices realized the highest efficiency of 17.1% (Supplementary Fig. 10b, c and Supplementary Table 6). These results demonstrate the universality of polyfullerene guests in increasing PCEs of all-PSCs.

## Discussion

In this work, we successfully introduce a poly(fullerene-*alt*-xylene) acceptor (PFBO-C12) as the guest component for high-performance all-PSCs. TAS characterization unveils that PFBO-C12 facilitates the hole transfer from PY-V-*γ* to PM6 and undergoes FRET with PY-V-*γ*. TPV/TPC results show that PFBO-C12 suppresses the charge recombination in the all-PSCs. Morphological investigations reveal that the incorporation of PFBO-C12 enhances the film crystallinity and reduces phase segregation simultaneously. Owing to these beneficial effects, the introduction of PFBO-C12 as the guest component to the PM6:PY-V-*γ* host blend eventually realizes high PCEs of 18.0% and 14.6% in rigid and flexible all-PSCs, respectively. Meanwhile, PFBO-C12-based ternary all-PSCs achieve both better light stability and bending durability compared to their PCBM counterparts. In conclusion, our work demonstrates that employing polyfullerene-type materials as guest components is an effective strategy for high-performance all-PSCs with better efficiencies, robustness, and stability.

## Methods

### Material synthesis and characterizations

PY-V-*γ* was synthesized according to our previous work. PM6 was purchased from Solarmer Energy Inc. Toluene was freshly distilled from sodium prior to use and benzophenone was used as the indicator. All other reagents and chemicals were purchased from commercial sources and used without further purification^1^H and ^13^C NMR spectra were obtained with a Bruker AV-400 MHz NMR spectrometer. Chemical shifts were reported in parts per million (ppm, δ)^1^ H, and ^13^C NMR spectra were referenced to tetramethylsilane (0 ppm) in CDCl_3_. Mass spectra were collected on a MALDI Micro MX mass spectrometer, or an API QSTAR XL System.

### Molecular weight measurement

The molecular weight of PFBO-C12 was measured using a high-temperature gel permeation chromatography (HT-GPC, Agilent PL-GPC220) at 150 °C with 1,2,4-trichlorobenzene as the eluent and polystyrenes as the standard.

### Optical characterizations

Film UV–Vis absorption spectra were acquired on a Perkin Elmer Lambda 20 UV/VIS Spectrophotometer. All film samples were spin-cast on ITO substrates.

### Electrochemical characterizations

Cyclic voltammetry was carried out on a CHI610E electrochemical workstation with the three electrodes configuration, using Ag/AgCl as the reference electrode, a Pt plate as the counter electrode, and a glassy carbon as the working electrode. 0.1 mol L^−1^ tetrabutylammonium hexafluorophosphate in anhydrous acetonitrile was used as the supporting electrolyte. The polymer and small molecules were drop-cast onto the glassy carbon electrode from chloroform solutions (5 mg/mL) to form thin films. Potentials were referenced to the ferrocenium/ferrocene couple by using ferrocene as external standards in acetonitrile solutions. The scan rate is 100 mV s^−1^. The conversion of reduction/oxidation onsets and LUMO/HOMO energy levels can be described as: equation (2): *E*_LUMO_ = −[*e*(*E* ^red^–*E* ^Fc/Fc+^)+4.8]; equation (3): *E*_HOMO_ = −[*e*(*E* ^ox^–*E* ^Fc/Fc+^)+4.8].

### Solar cell fabrication and testing

OSCs were made followed a device structure of glass/ITO (indium tin oxide)/PEDOT:PSS(poly(3,4-ethylenedioxythiophene):poly(styrene sulfonate))/PM6:acceptor/PNDIT-F3N ([(9,9-bis(3’-(N,N-dimethylamino)propyl)−2,7-fluorene)-alt-5,5’-bis(2,2’-thiophene)−2,6-naphthalene-1,4,5,8-tetracaboxylic-N,N’-di(2-ethylhexyl)imide])/Ag. The patterned ITO-coated glass substrates were cleaned in detergent, de-ionized water, acetone, and isopropanol sequentially by ultra-sonic bath for 30 min, respectively, and then dried in an oven at 70 °C overnight. Further UV-Ozone treatment for 20 min was applied before use to improve its wettability. Then the PEDOT:PSS (Heraeus Clevios P VP. AI 4083, filtered at 0.45 μm) was spin-coated onto the cleaned ITO-coated glass substrate at 5200 rpm for 30 s followed by annealing at 150 °C for 15 min in the air to obtain ~30 nm thick film. The PEDOT:PSS-coated ITO substrates were then transferred into an N_2_-filled glove box for further device fabrication. PM6 was dissolved in chlorobenzene (CB, the concentration of donor was 9 mg mL^−1^) and stirred at 60 °C for 5 h to form a donor solution. The acceptor materials were dissolved in chloroform (CF, the concentration of acceptor was 9 mg mL^−1^) with the solvent additive of CN (1.5% of volume) with/without PCBM and PFBO-C12 followed by stirring at room temperature (RT) for 1 h to form the acceptor solution. The amount of PFBO-C12 was screened and the optimized ratio of PFBO-C12 is 10% (Table S7). For sequential deposition (SD)-based devices, PM6 solution was spun followed by the solution of the acceptor, and then the active layer was annealed at 100 °C for 5 min via SD processing. The active layer thickness is ~120 nm measured by the Bruker Dektak XT profilometer. A thin layer of PNDIT-F3N (~10 nm) was spin-coated onto the active layer, and Ag electrode (~100 nm) was deposited on top of the electron transfer layer in a thermal evaporator under a vacuum of 1 × 10^−5^ Pa through a shadow mask. The effective device area was 4.0 mm^2^ defined by a metal mask with an aperture aligned with the device area. The current–voltage (*J*–*V*) characteristics of the photovoltaic devices were measured by a Keithley 2400 Source Meter under RT in the glove box. The photocurrent was measured under AM 1.5 G illumination at 100 mW cm^−2^ using a standard Si solar cell (with KG5 filter) and a readout meter to calibrate the light intensity.

### EQE measurements

EQE spectra were measured using an Enlitech QE-S EQE system equipped with a standard Si diode. Monochromatic light was generated from a Newport 300 W lamp source.

### TR-PL characterizations

TR-PL measurements were carried out on encapsulated thin films. A 100 fs Ti:Sapphire oscillator (Coherent Mira 900) operating at 76 MHz repetition rate was tuned to 750 nm and focused to excite the sample. The PL was collected and guided into a spectrometer equipped with a silicon single-photon counter to carry out time-correlated single-photon counting (TCSPC), integrating the majority of the PL. Low-temperature measurements were carried out with the sample mounted inside a helium flow cryostat with an active feedback temperature controller. The PL spectra were obtained using a Si photodiode array detector.

### Exciton dissociation probability analysis

Exciton dissociation (*P*_diss_) and collection (*P*_coll_) efficiencies can be calculated from the relationships between photocurrent (*J*_ph_) and effective voltage (*V*_eff_). *J*_ph_ is defined as the difference between the dark current density (*J*_D_) and the current density under illumination (*J*_L_). The definition of *V*_eff_ is the absolute value of *V*_0_−*V*_appl_, where *V*_0_ refers to the voltage value when *J*_L_ = *J*_D_ and *V*_appl_ is the applied voltage. At high *V*_eff_, almost all excitons are separated and extracted, and *J*_ph_ reaches saturation (*J*_sat_). Accordingly, *η*_diss_ and *η*_coll_ can be determined by *J*_SC_/*J*_sat_ and *J*_max_/*J*_sat_, respectively, in which *J*_max_ is the current density at the maximal power output point.

### TPV and TPC characterizations

The device samples were mounted on a conductive clip and under steady-state illumination from a focused Quartz Tungsten-Halogen Lamp light source. The measurements were performed with a background response similar to open-circuit voltage. An optical perturbation was applied to the devices with a 1 kHz femtosecond pulse laser under 600 nm excitation. The TPV signal was acquired by a digital oscilloscope at open-circuit conditions. TPC signal was measured under approximately short-circuit conditions with 50 Ω impedance. The intensity-dependent TPV and TPC measurements were performed by applying various neutral density filters to tune down the background white light and 750 nm pulse excitation. The TPV lifetime *τ* was fitted with the monoexponential model under a small perturbation approximation. The charges generated Δ*Q* by the pulse laser were calculated by the integrated TPC peak. The differential capacitance *C* is then calculated by equation (4): *C* = Δ*Q*/Δ*V*_0_, where the Δ*V*_0_ is the amplitude of the pulse-generated TPV signal at the corresponding experimental condition. *C* was fitted with exponential models as a function of *V*_OC_ and then integrated with respect to voltage to acquire the carrier density. The TPV lifetime *τ* under different carrier densities *n* was then fitted to a power law with exponent *λ*^55,56^.

### Hole mobility measurement

The hole mobilities were measured using the space charge limited current (SCLC) method, employing a device architecture of ITO/PEDOT:PSS/blend film/MoO_3_/Ag. The mobilities were obtained by taking current–voltage curves and fitting the results to a space charge limited form, where the SCLC is described by equation (5):

$$J=\frac{9{\varepsilon }_{0}{\varepsilon }_{{{{{{\rm{r}}}}}}}\mu {({V}_{{{{{{\rm{appl}}}}}}}-{V}_{{{{{{\rm{bi}}}}}}}-{V}_{{{{{{\rm{s}}}}}}})}^{2}}{8{L}^{3}}$$

where *ε*_0_ is the permittivity of free space, *ε*_r_ is the relative permittivity of the material (assumed to be 3), *μ* is the hole mobility, *L* is the thickness of the film, *V*_appl_ is the applied voltage, *V*_bi_ is the built-in voltage (0.15 V) and *V*_s_ is the voltage drop from the substrate’s series resistance (*V*_s_ = *IR*, *R* was measured to be 10.8 Ω). From the plots of *J*^1/2^ vs. *V*_appl_–*V*_bi_–*V*_s_, hole mobilities can be deduced.

### Electron mobility measurement

The electron mobilities were measured using the SCLC method, employing a device architecture of ITO/ZnO/blend film/PNDIT-F3N/Ag. The mobilities were obtained by taking current-voltage curves and fitting the results to a space charge limited form, where the SCLC is described by equation (6):

$$J=\frac{9{\varepsilon }_{0}{\varepsilon }_{{{{{{\rm{r}}}}}}}\mu {({V}_{{{{{{\rm{appl}}}}}}}-{V}_{{{{{{\rm{bi}}}}}}}-{V}_{{{{{{\rm{s}}}}}}})}^{2}}{8{L}^{3}}$$

where *ε*_0_ is the permittivity of free space, *ε*_r_ is the relative permittivity of the material (assumed to be 3), *μ* is the electron mobility, *L* is the thickness of the film, *V*_bi_ is the built-in voltage (0.15 V) and *V*_s_ is the voltage drop from the substrate’s series resistance (*V*_s_ = *IR*, *R* was measured to be 10.8 Ω). From the plots of *J*^1/2^ vs. *V*_appl_–*V*_bi_–*V*_s_, electron mobilities can be deduced.

### GIWAXS characterization

Grazing-incidence wide-angle X-ray scattering (GIWAXS) measurements were conducted at Advanced Light Source (ALS), Lawrence Berkeley National Laboratory, Berkeley, CA at beamline 7.3.3. Data were acquired at the critical angle (0.13°) of the film with a hard X-ray energy of 10 keV. X-ray irradiation time was 10 s, dependent on the saturation level of the detector. Samples were prepared on Si substrates using identical blend solutions as those used in devices. The coherence length was calculated using the Scherrer equation (7): CL = 2*πK*/Δ*q*, where Δ*q* is the full-width at half-maximum of the peak and *K* is a shape factor (1 was used here).

### GISAXS characterization

The grazing incidence small-angel X-ray scattering (GISAXS) measurements were carried out with a Xeuss 2.0 SAXS/WAXS laboratory beamline using a Cu X-ray source (8.05 keV, 1.54 Å) and a Pilatus3R 300 K detector. The incident angle was 0.2°.

### FTPS-EQE characterization

Fourier-transform photocurrent spectroscopy external quantum efficiency (FTPS-EQE) spectra were measured by using a Vertex 70 from Bruker optics and a QTH lamp. The electroluminescence signal was collected with a monochromator and detected with a Si-CCD detector.

### TAS characterization

Two beams were split from the 800 nm output of a Ti:Sapphire laser amplifier (Coherent Legend Elite, repetition rate of 1 kHz, 100 fs). One of the split outputs was used to photoexcite the sample either directly at 800 nm or at other wavelengths generated in an optical parametric amplifier (Coherent OPerA Solo). The other split output was used to generate a white light continuum probe pulse by focusing the beam onto a sapphire plate. The optical pulses were spatially overlapped in the encapsulated thin-film sample and temporally delayed using a motor-driven delay stage. The probe was guided into a spectrometer (Acton instruments SpectraPro 275) with a 50 LP/mm grating and detected by a Si array detector. The pump-induced change in transmission (*∆*T/T) was recorded shot by shot. The experiment was performed with pump fluence of ~2 µJ cm^−2^ per pulse. Background signals were subtracted from the signal, and the chirp response was corrected. For the hole transfer study, a pump of 800 nm was used to selectively photoexcite the acceptor part. For the fullerene excitation, a pump of 370 nm was used to preferentially excite the guest components.

### Reporting summary

Further information on research design is available in the Nature Portfolio Reporting Summary linked to this article.

## Supplementary information

Supplementary Information

Reporting Summary

## Data Availability

All data generated in this study are included in this published article and its Supplementary Information, and the raw data generated in this study are provided in the Source Data file. Source data are provided with this paper.

## Source data

Source Data
