# Supplementary material for: Improved photovoltaic performance and robustness of all-polymer solar cells enabled by a polyfullerene guest acceptor
Source: Nat Commun. 2023 Apr 22;14:2323. doi: 10.1038/s41467-023-37738-9 (PMC10122667; doi:10.1038/s41467-023-37738-9)
Supplement: Supplementary file 1 — Supplementary Information [file 41467_2023_37738_MOESM1_ESM.pdf]

Improved Photovoltaic Performance and Robustness of All-Polymer Solar Cells Enabled by A Polyfullerene Guest Acceptor

Han Yu[1,2,#], Yan Wang[3,#], Xinhui Zou[2,4,#], Junli Yin[1,2], Xiaoyu Shi[1], Yuhao Li[5], Heng Zhao[6], Lingyuan Wang[1], Ho Ming Ng[2], Bosen Zou[2], Kam Sing Wong[4]*, Xinhui Lu[5], Wei Ma[6], Zonglong Zhu,[3]* He Yan[2]* & Shangshang Chen[1]*

[1]State Key Laboratory of Coordination Chemistry, MOE Key Laboratory of High-Performance Polymer Materials & Technology, School of Chemistry and Chemical Engineering, Nanjing University, Nanjing, Jiangsu 210023, China
E-mail: schen@nju.edu.cn

[2]Department of Chemistry, Guangdong-Hong Kong-Macao Joint Laboratory of Optoelectronic and Magnetic Functional Materials, Energy Institute and Hong Kong Branch of Chinese National Engineering Research Center for Tissue Restoration & Reconstruction, Hong Kong University of Science and Technology, Clear Water Bay, Kowloon, Hong Kong 999077, China.
E-mail: hyan@ust.hk

[3]Department of Chemistry and Hong Kong Institute for Clean Energy, City University of Hong Kong, Kowloon, Hong Kong 999077, China
Email: zonglzhu@cityu.edu.hk

[4]Department of Physics, Hong Kong University of Science and Technology, Clear Water Bay, Kowloon, Hong Kong 999077, China
Email: phkswong@ust.hk

[5]Department of Physics, Chinese University of Hong Kong, New Territories, Hong Kong 999077, China

[6]State Key Laboratory for Mechanical Behavior of Materials, Xi'an Jiaotong University, Xi'an 710049, China

[#]These authors contributed equally.

Table of Contents

1. **Supplementary Figures**······2

2. **Supplementary Tables**······17

3. **Supplementary Methods**······22

4. **Supplementary References**······24

1. Supplementary Figures

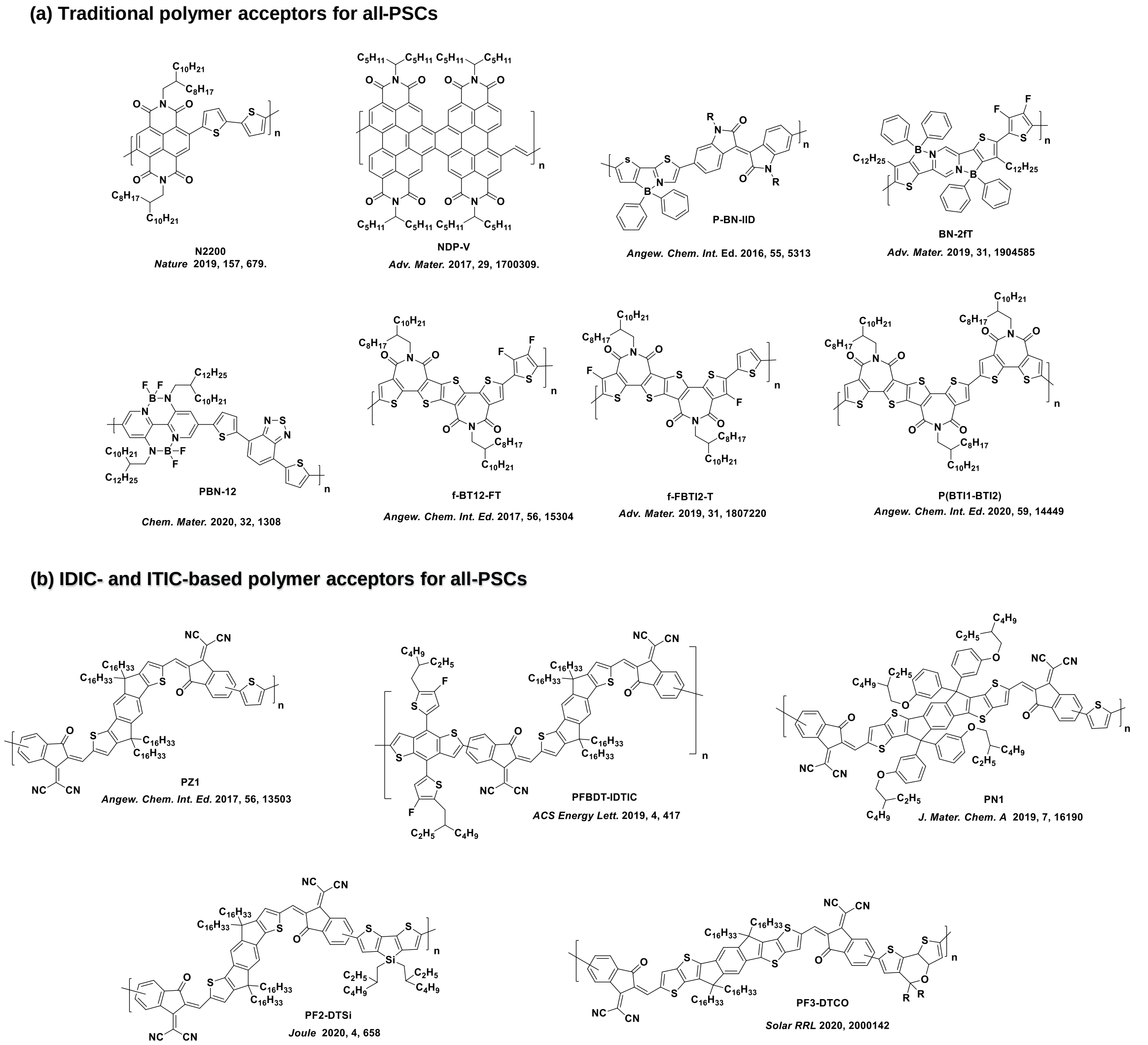

Supplementary Figure 1. Molecular structures of the traditional polymer acceptors in all-PSCs. (a) naphthalene diimide, perylene diimide, B←N unit and bithiophene imide-based polymer acceptors. (b) Representative molecular structures of the IDIC- and ITIC-based polymer acceptors for all-PSCs.

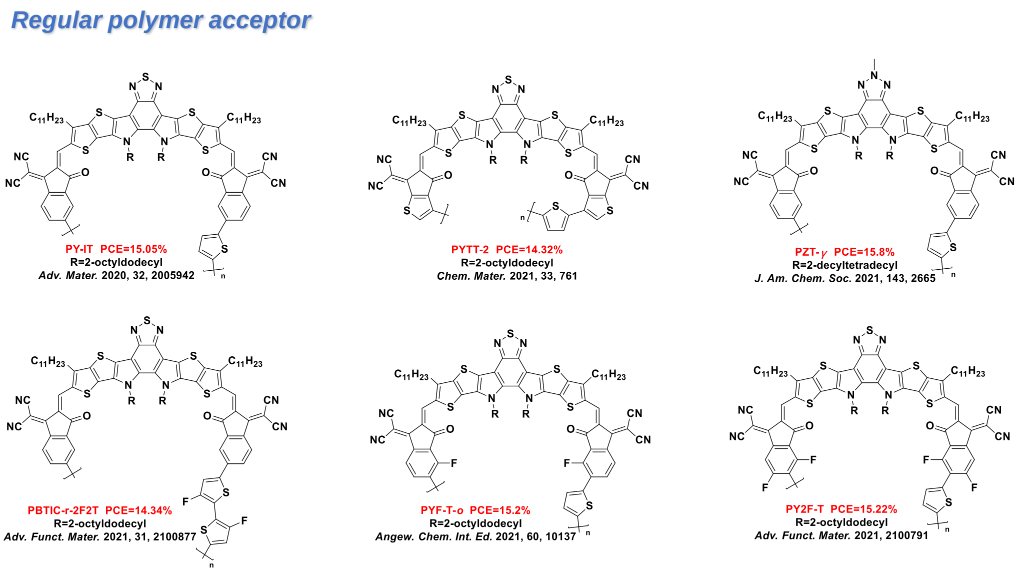

Supplementary Figure 2. **Molecular structures of Y-series regular polymer acceptors.** Recent reported regular polymer acceptors and the corresponding PCEs in the binary all-PSCs.

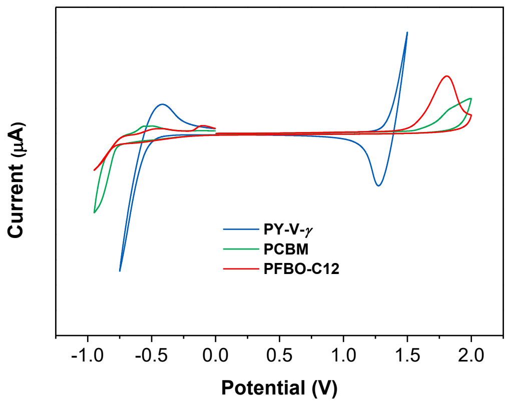

Supplementary Figure 3. Electrochemical measurement. Cyclic voltammetry curves of PY-V-γ, PCBM, and PFBO-C12.

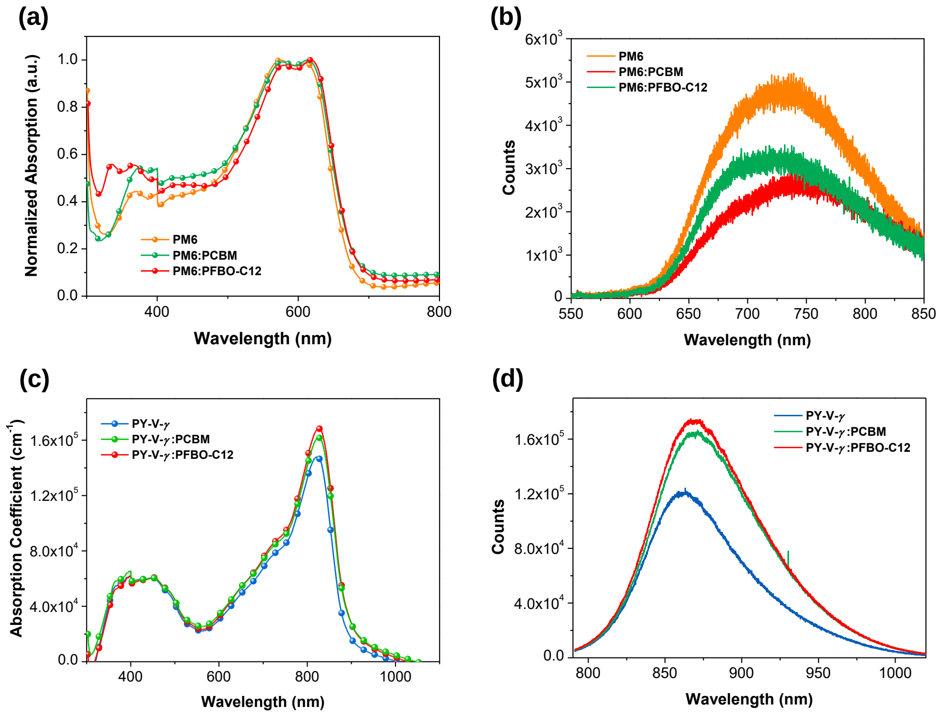

Supplementary Figure 4. Absorption and Emission properties. (a) Normalized absorption and (b) photoluminescence spectra of PM6, PM6:PCBM and PM6:PFBO-C12 films. (c) Absorption coefficient and (d) photoluminescence spectra of PY-V-γ, PY-V-γ:PCBM and PY-V-γ:PFBO-C12 films. The discontinuity in (a) and (c) is due to the normal switching of the light source from the tungsten lamp to the deuterium lamp at 400 nm.

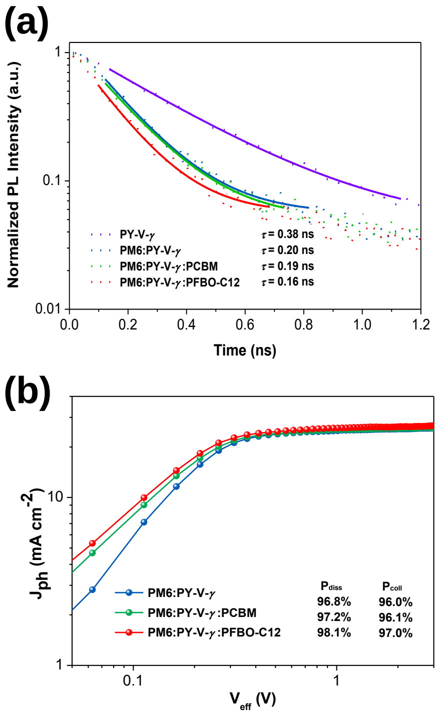

Supplementary Figure 5. **Exciton dissociation characterization.** (a) TR-PL decay curves of the PY-V-*γ* neat film, and the PM6:PY-V-*γ*, PM6:PY-V-*γ*:PCBM, and PM6:PY-V-*γ*:PFBO-C12 blend films. The excitation and probe wavelengths are 750 and 850 nm, respectively. (b) J_{ph}-J_{eff} curves of the all-PSCs based on PM6:PY-V-*γ*, PM6:PY-V-*γ*:PCBM, and PM6:PY-V-*γ*:PFBO-C12.

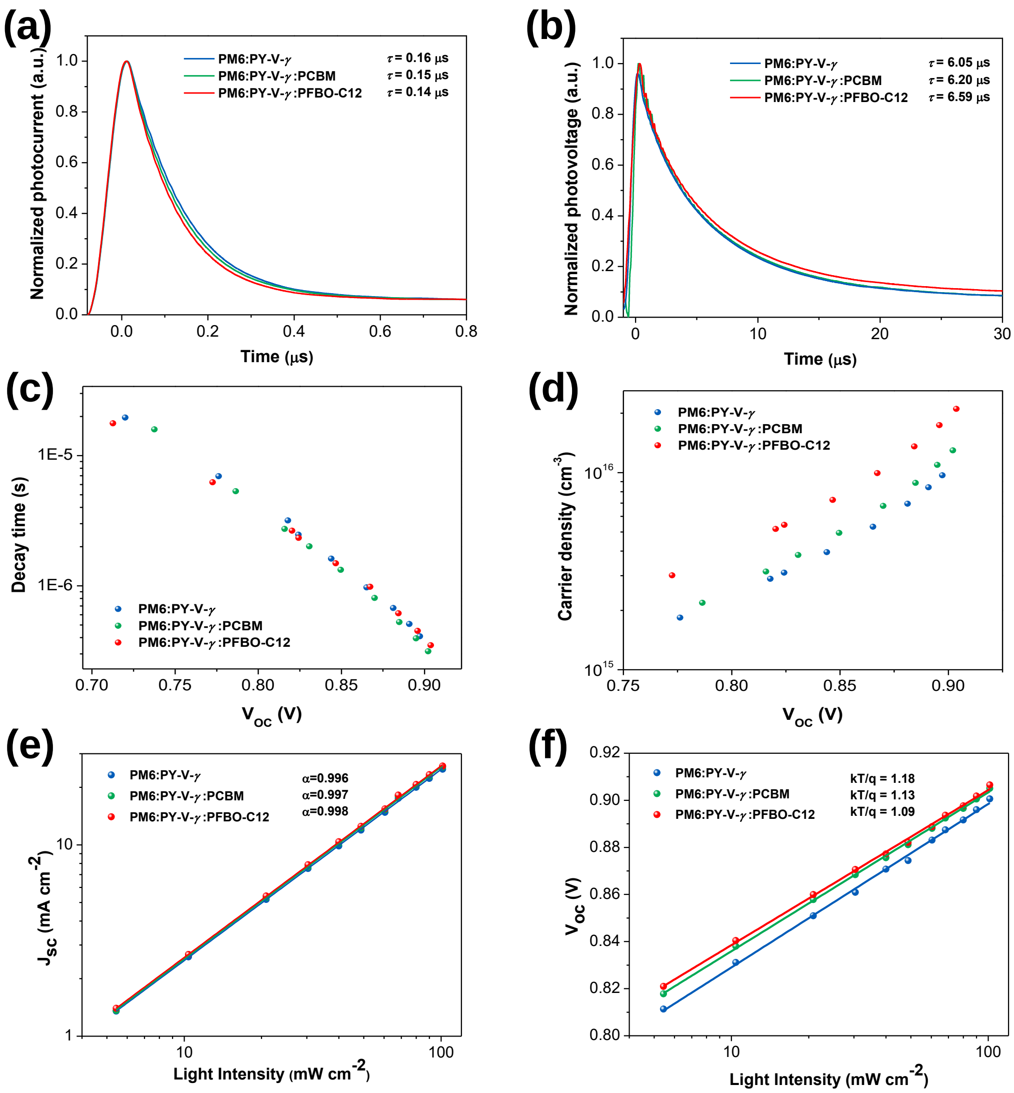

Supplementary Figure 6. Charge recombination characterization. (a) TPC and (b) TPV curves of the PM6:PY-V-γ, PM6:PY-V-γ:PCBM and PM6:PY-V-γ:PFBO-C12 based all-PSCs. (c) TPV decay time as a function of V_{OC}. (d) Calculated carrier density as a function of V_{OC} based on the three all-PSCs. (e) The J_{SC} and (f) V_{OC} on different light intensity (P_{light}) of the corresponding all-PSCs.

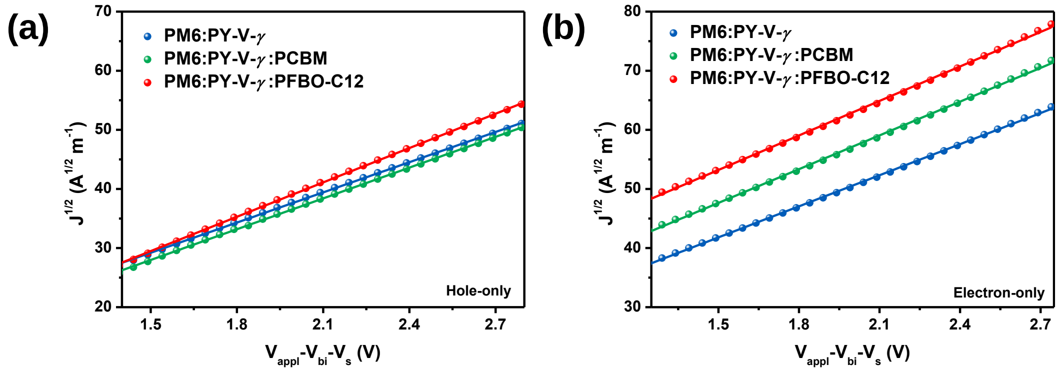

Supplementary Figure 7. Carrier mobility. $J^{1/2}{\sim}V$ characteristics of (a) hole-only devices and (b) electron-only devices based on PM6:PY-V-γ, PM6:PY-V-γ:PCBM, and PM6:PY-V-γ:PFBO-C12 blend films.

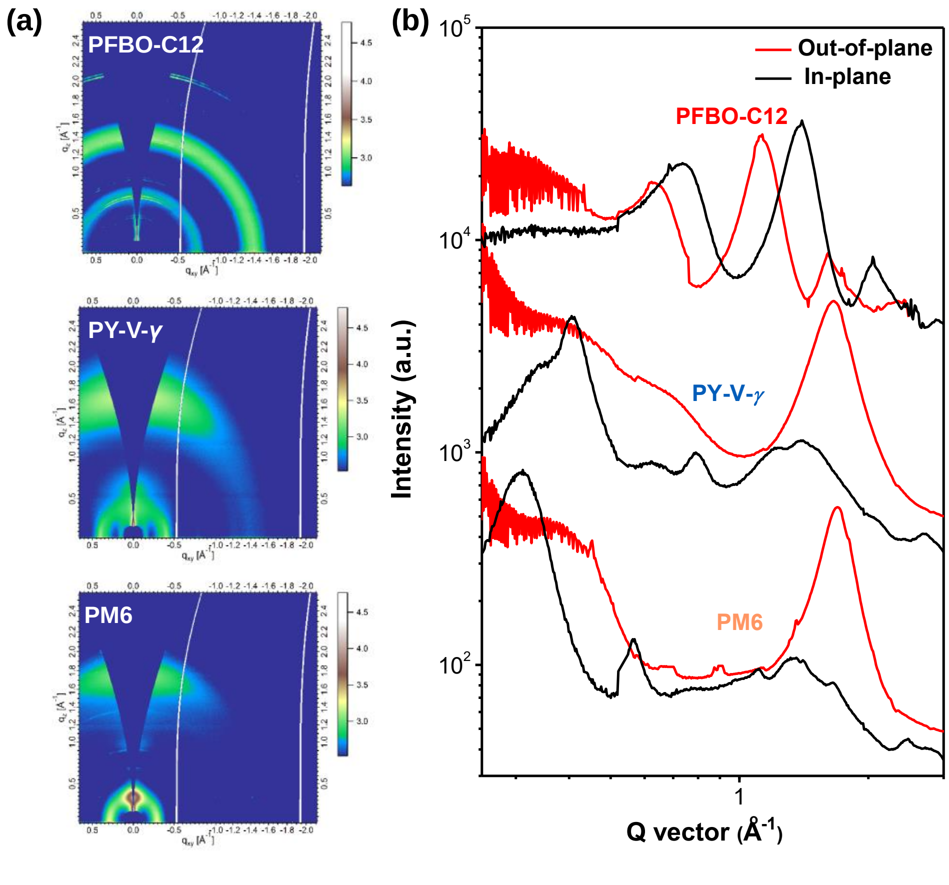

Supplementary Figure 8. **Morphology of pristine films.** (a) 2D GIWAXS patterns of PFBO-C12, PY-V-γ and PM6 neat films and (b) the corresponding 1D GIWAXS line-cuts of the in-plane and out-of-plane directions.

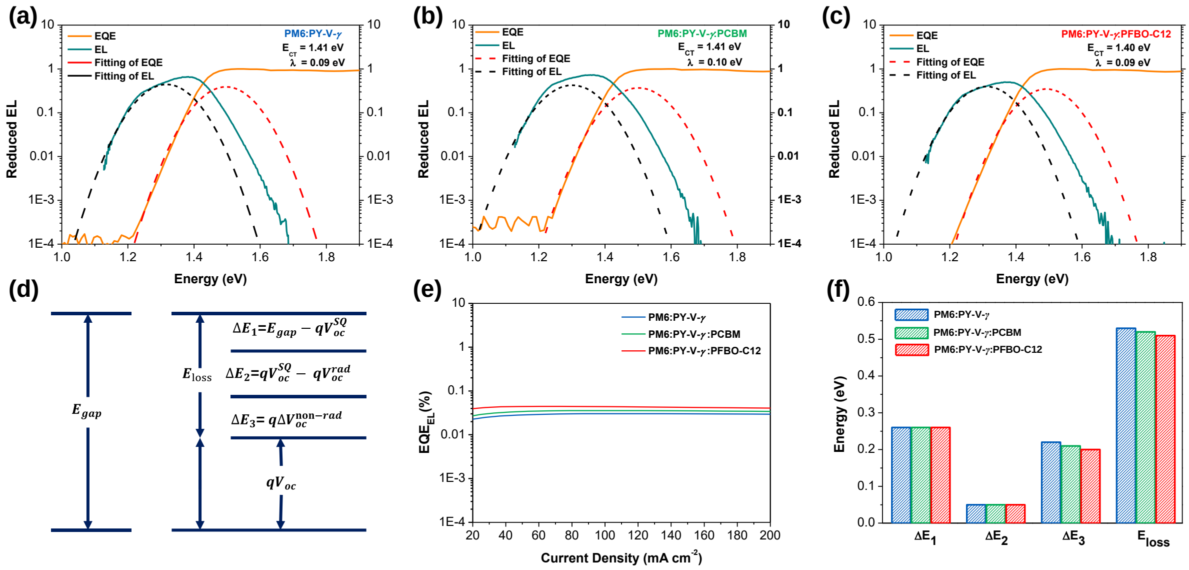

Supplementary Figure 9. Energy loss analysis. (a–c) Semilogarithmic plots of normalized electroluminescence (EL) and EQE calculated by Fourier transform photocurrent spectroscopy (EQE_{FTPS}) as a function of energy for the devices based on PM6:PY-V-*γ*, PM6:PY-V-*γ*:PCBM, and PM6:PY-V-*γ*:PFBO-C12. (d) The schematic diagram for energy losses (E_{loss}) of OSCs according to the detailed balance theory. (e) EL quantum efficiency as dependence on current density. (f) Quantitative analysis results of E_{loss}.

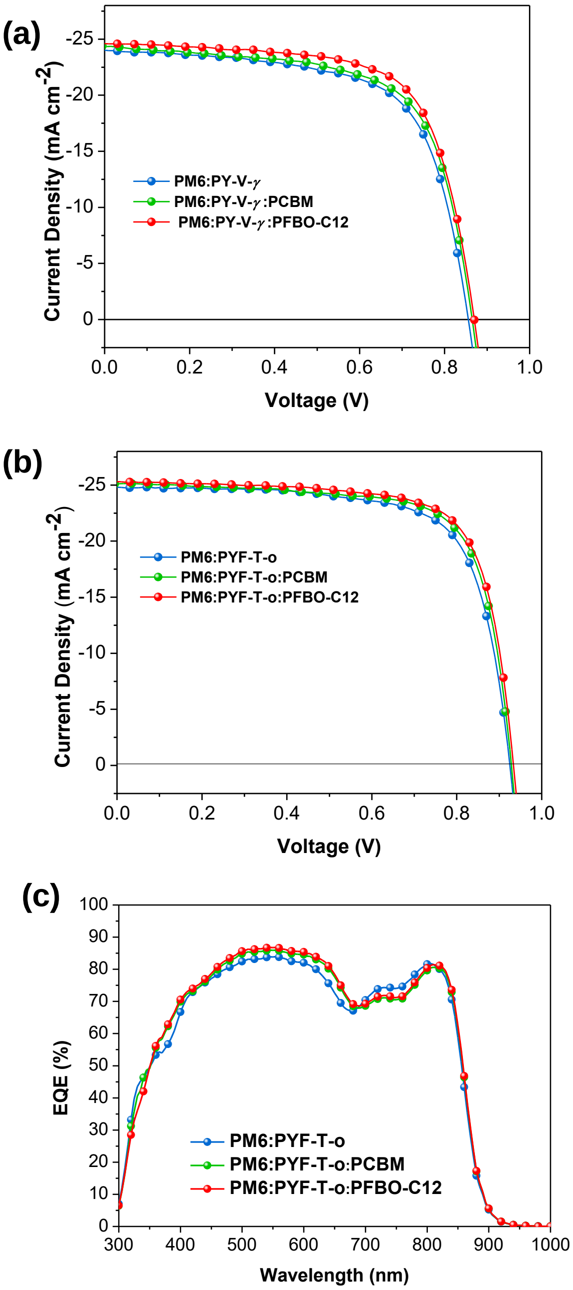

Supplementary Figure 10. Flexible device performance and universality in other systems. (a) *J-V* characteristic curves of the three flexible all-PSCs based on PM6:PY-V-*γ*, PM6:PY-V-*γ*:PCBM and PM6:PY-V-*γ*:PFBO-C12. (b) *J-V* characteristic curves of all-PSCs based on PM6:PYF-T-*o*, PM6:PYF-T-*o*:PCBM and PM6:PYF-T-*o*:PFBO-C12. (c) EQE curves of the devices based on PM6:PYF-T-*o*, PM6:PYF-T-*o*:PCBM and PM6:PYF-T-*o*:PFBO-C12.

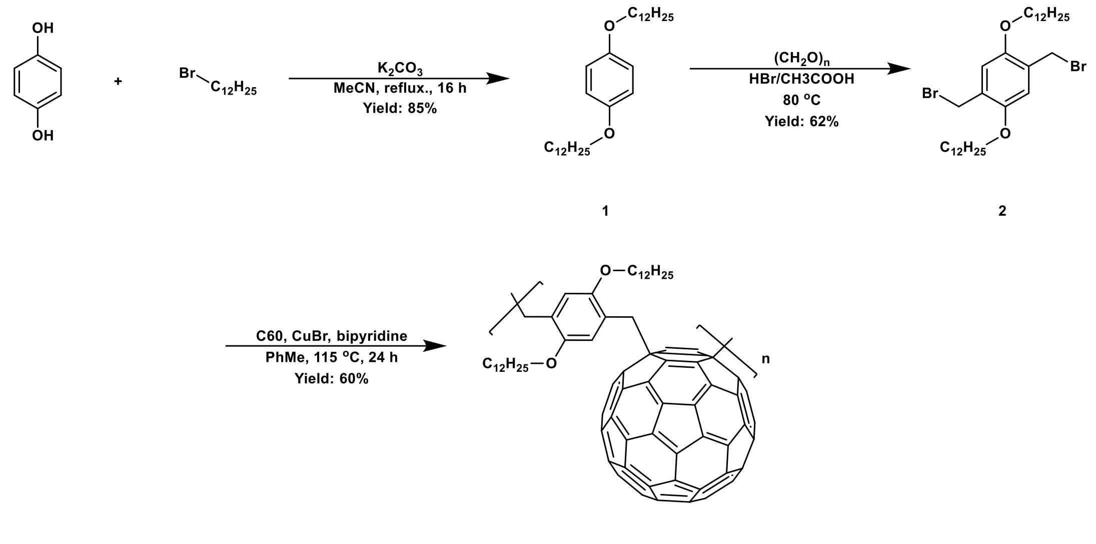

Supplementary Figure 11. Synthesis route of PFBO-C12.[1]

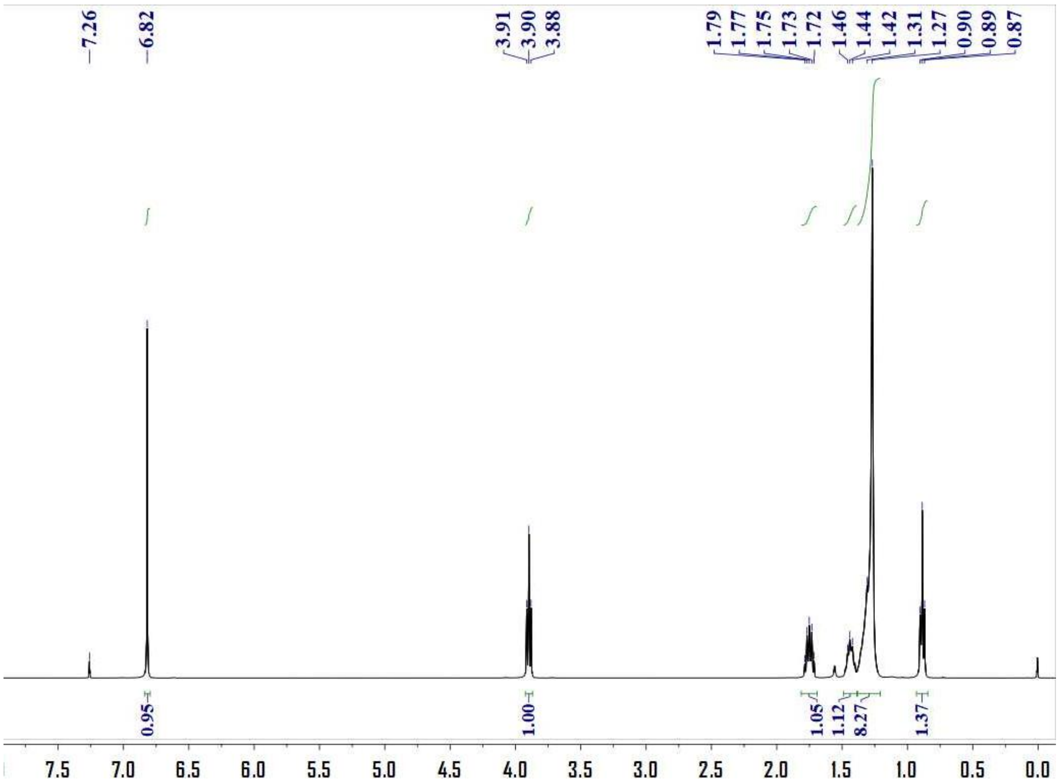

Supplementary Figure 12. ^{1}HNMR spectrum of Compound 1 (400 MHz, $CDCl_3$).

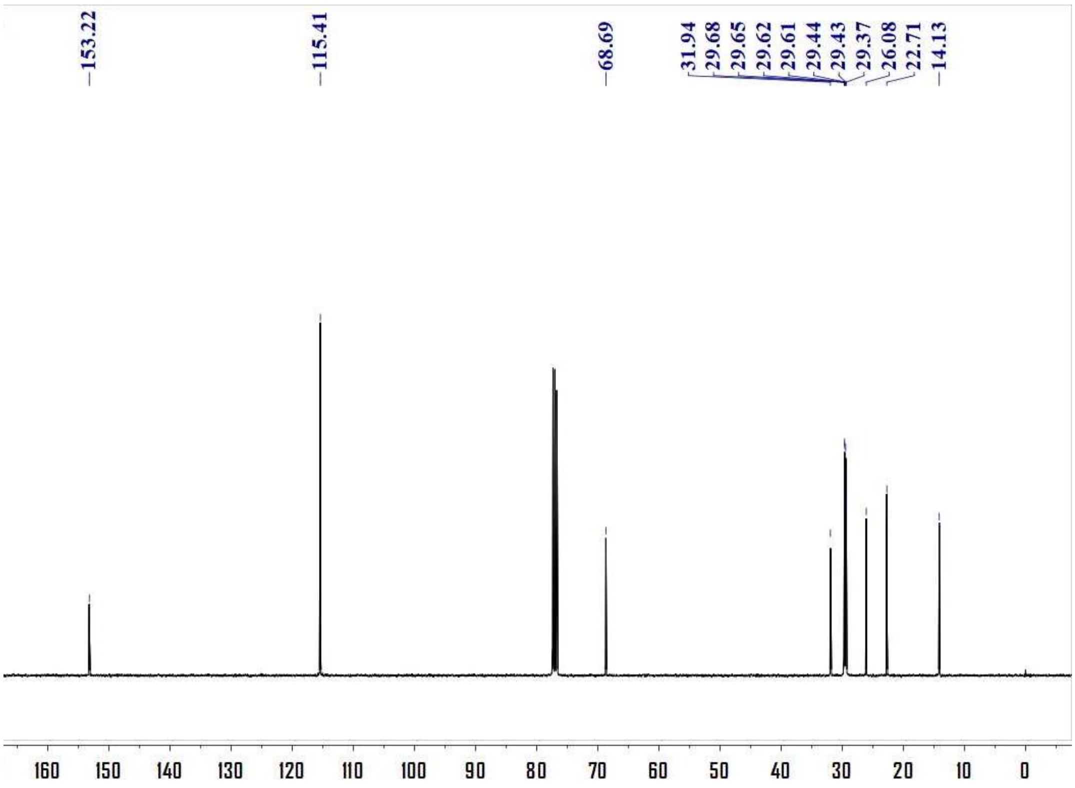

Supplementary Figure 13. ^{13}CNMR spectrum of Compound 1 (100 MHz, $CDCl_3$).

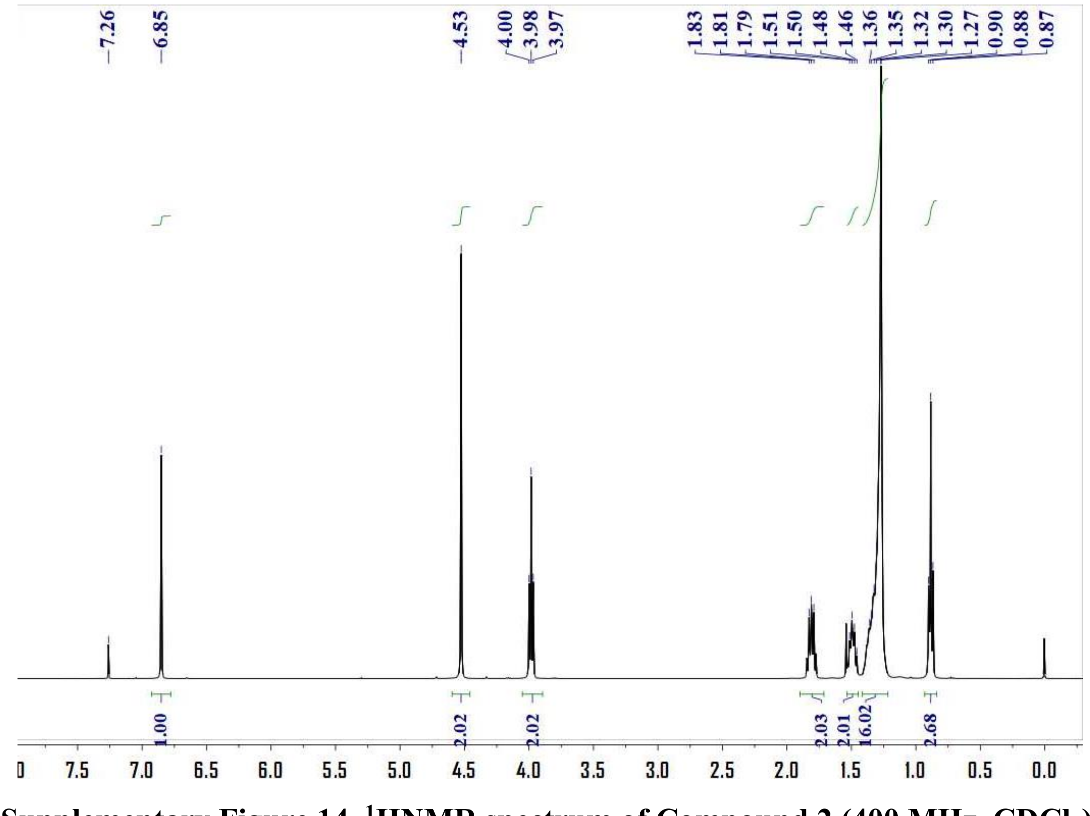

Supplementary Figure 14. ^{1}HNMR spectrum of Compound 2 (400 MHz, $CDCl_3$).

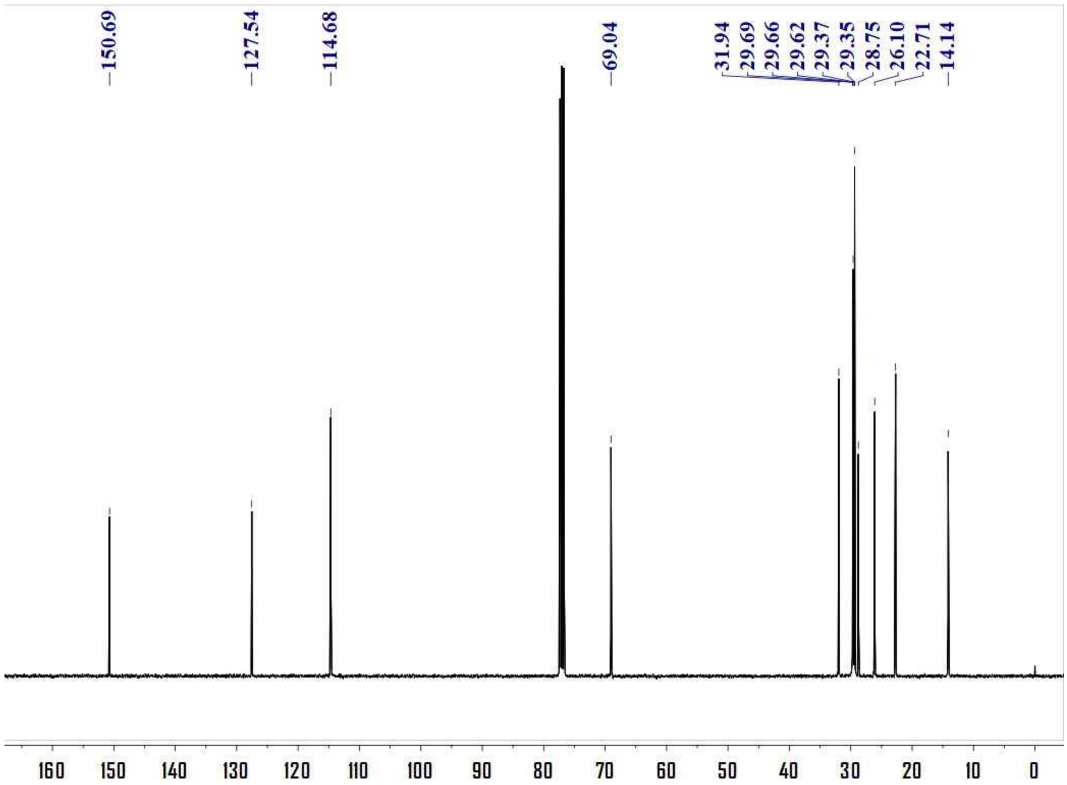

Supplementary Figure 15. ^{13}CNMR spectrum of Compound 1 (100 MHz, $CDCl_3$).

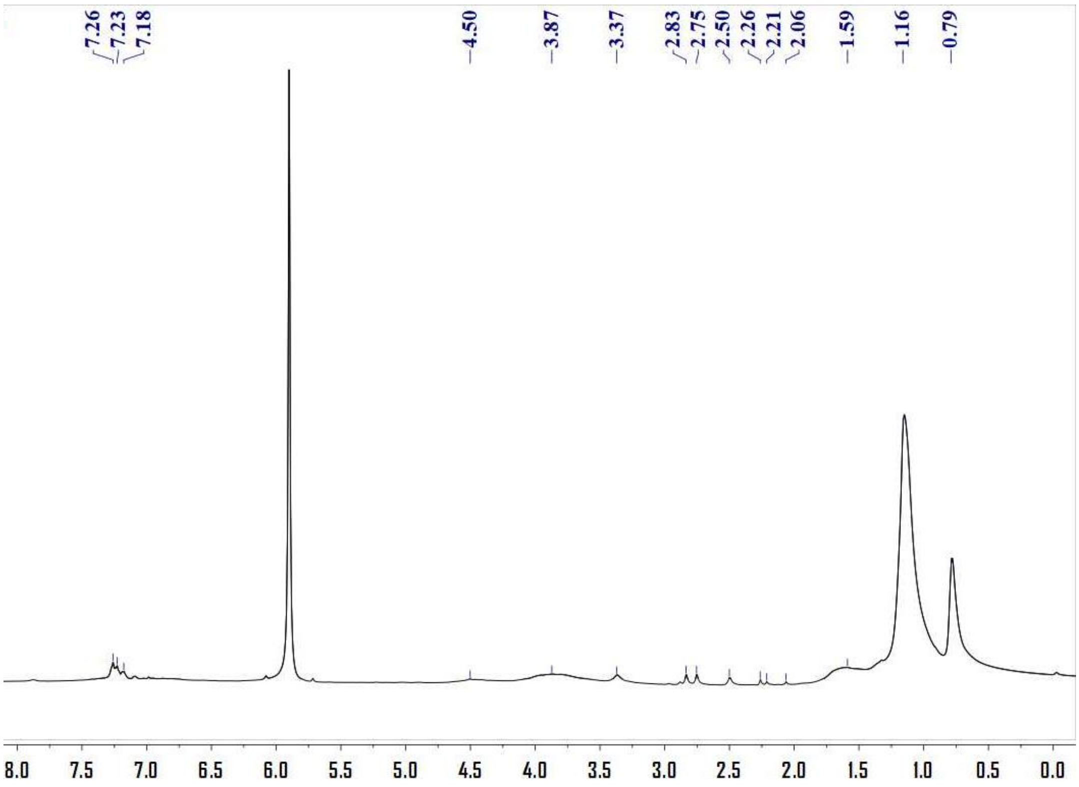

Supplementary Figure 16. ^{1}HNMR spectrum of PFBO-C12 (400 MHz, $CDCl_3$).

Molecular Weight Averages

Peak	Mp (g/mol)	Mn (g/mol)	Mw (g/mol)	Mz (g/mol)	Mz+1 (g/mol)	Mv (g/mol)	PD
Peak 1	15211	8802	18947	34787	55239	32172	2.153

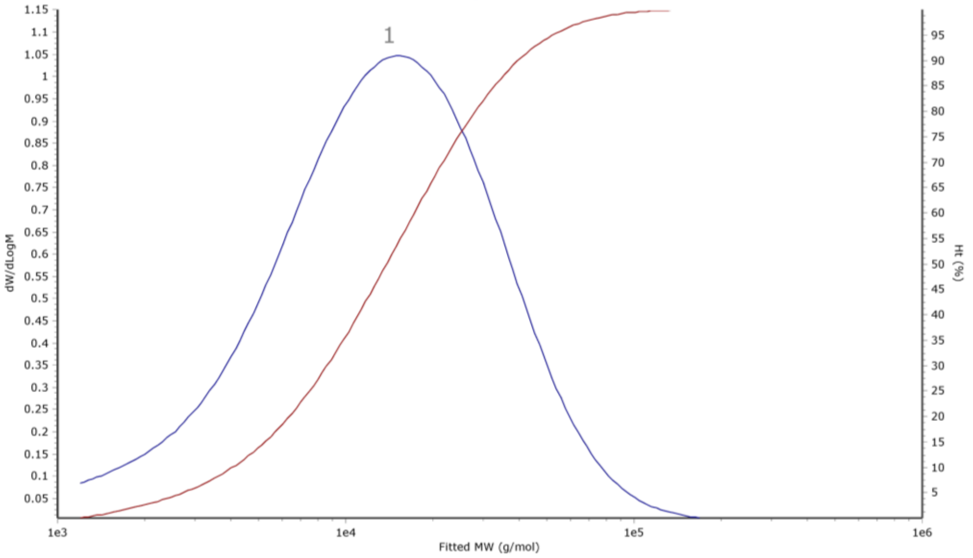

Supplementary Figure 17. GPC curve of PFBO-C12.

2. Supplementary Tables

Supplementary Table 1. Summary of photovoltaic performance parameters of the all-PSCs based on Y-series polymer acceptors.

	V_{oc} [V]	J_{sc} [mA cm^{-2}]	FF [%]	PCE [%]	Ref.
**PM6: PY-V-*γ*:PCBM**	**0.904**	**25.5**	**76.1**	**17.5**	**This work**
**PM6: PY-V-*γ*:PFBO-C12**	**0.905**	**25.8**	**77.0**	**18.0**	**This work**
PBDB-T:PJ1	0.90	22.6	71	14.4	[2]
PM6:PYT	0.93	21.78	66.33	13.44	[3]
PM6:PF3-DTCO	0.943	15.75	68.2	10.13	[4]
PBDB-T:PTPBT-$ET_{0.3}$	0.899	21.33	65.3	12.53	[5]
PM6:L14	0.96	20.6	72.1	14.3	[6]
PBDB-T:PF5-Y5	0.946	20.65	74.0	14.45	[7]
PM6:PY-IT	0.933	22.30	72.3	15.05	[8]
PTzBI-oF:PFA1	0.87	23.96	72.67	15.11	[9]
PM6:PYF-T	0.89	23.1	68.0	14.0	[10]
PM6:PYF-T-*o*	0.90	23.3	72.4	15.2	[11]
PBDB-T_{MW}:PJ1	0.90	22.2	75.3	15.4	[12]
PM6:PYTT-2	0.91	22.00	71.53	14.32	[13]
PBDB-T:PYN-BDTF	0.86	22.28	69	13.22	[14]
PBDB-T:PZT-*ϒ*	0.90	24.7	71.3	15.8	[15]
JD40:PJ1	0.91	23.2	75	15.8	[16]
PM6:PY-IT:BN-T	0.96	22.65	74.3	16.09	[17]
PM6:PY2F-T	0.86	24.27	72.62	15.22	[18]
PBDB-T:PN-Se	0.907	24.82	71.8	16.16	[19]
JD40:PJTVT	0.89	23.75	76.40	16.13	[20]
PBDB-T:PY-Se	0.891	23.52	73.85	15.48	[21]
PBDB-T:PFY-3Se	0.871	23.6	73.7	15.1	[22]

PM6:PY2Se-Cl	0.884	24.5	74.3	16.1	[23]
PM6:PYT-1S1Se	0.926	24.1	73.0	16.3	[24]
PBDB-T:PTCl$_o$-Y	0.948	20.21	66.5	12.74	[25]
PBDB-T:PY5-BTZ	0.922	22.61	71.1	14.82	[26]
PBDB-T: Y5-Se-In	0.86	21.74	72.0	12.92	[27]
PM6:PY-DT	0.949	23.58	74.4	16.76	[28]
JD40:PA-5	0.87	24.05	76.88	16.11	[29]
PBDB-T:PYTS-0.3	0.92	22.91	70	14.68	[30]

Supplementary Table 2. The parameters of electron mobilities and hole mobilities of PM6:PY-V-γ, PM6:PY-V-γ:PCBM and PM6:PY-V-γ:PFBO-C12.

Active Layer	μ_e (cm^2 V^{-1} s^{-1})	μ_h (cm^2 V^{-1} s^{-1})
PM6:PY-V-γ	$(9.0\pm0.6)\times10^{-4}$	$(8.6\pm0.6)\times10^{-4}$
PM6:PY-V-γ:PCBM	$(1.1\pm0.1)\times10^{-3}$	$(9.1\pm0.7)\times10^{-4}$
PM6:PY-V-γ:PFBO-C12	$(1.2\pm0.1)\times10^{-3}$	$(1.1\pm0.1)\times10^{-3}$

Supplementary Table 3. **Morphology parameters extracted from the GIWAXS measurements.**

Sample	π-π stacking distance (010)		π-π stacking coherence (010)	
	q_z (Å^{-1})	$d_{\pi-\pi}$ (Å)	w (Å^{-1})	CL (Å)
PM6:PY-V-γ	1.70	3.70	0.25	27
PM6:PY-V-γ:PCBM	1.71	3.68	0.28	25
PM6:PY-V-γ:PFBO-C12	1.71	3.68	0.25	27
PFBO-C12	1.38	4.55	0.19	39
PY-V-γ	1.66	3.79	0.25	28
PM6	1.74	3.62	0.24	28
**Sample**	**lamellar stacking distance (100)**		**lamellar stacking coherence (100)**	
	q_r (Å^{-1})	$d_{\pi-\pi}$ (Å)	w (Å^{-1})	CL (Å)
PM6:PY-V-γ	0.31	20.5	0.094	74
PM6:PY-V-γ:PCBM	0.31	20.5	0.095	74
PM6:PY-V-γ:PFBO-C12	0.31	20.5	0.087	80
PFBO-C12	0.74	8.5	0.175	40
PY-V-γ	0.34	18.3	0.066	106
PM6	0.30	21.2	0.078	90
**Sample**	**π-π stacking distance (200)**		**π-π stacking coherence (200)**	
	q_r (Å^{-1})	$d_{\pi-\pi}$ (Å)	w (Å^{-1})	CL (Å)
PM6:PY-V-γ	0.41	15.4	0.058	120
PM6:PY-V-γ:PCBM	0.42	15.1	0.057	122
PM6:PY-V-γ:PFBO-C12	0.41	15.4	0.051	137
PY-V-γ	0.41	15.4	0.052	136

Supplementary Table 4. Detailed E_{loss} parameters of the all-PSCs based on the three material systems.

Material Combination	E_g (eV)	qV_{oc}^{SQ} [a] (eV)	qV_{oc}^{rad} [b] (eV)	qV_{OC} (eV)	E_{loss} (eV)	ΔE_1 (eV)	ΔE_2 [c] (eV)	ΔE_3 [d] (eV)
**PM6:PY-V-*γ***	1.428	1.164	1.116	0.900	0.528	0.264	0.048	0.216
**PM6:PY-V-*γ*:PCBM**	1.425	1.161	1.114	0.904	0.521	0.264	0.047	0.210
**PM6:PY-V-*γ*:PFBO-C12**	1.420	1.155	1.106	0.905	0.514	0.265	0.048	0.201

[a] V_{oc}^{SQ} : Schokley-Queisser limit to V_{OC}.

[b] V_{oc}^{rad} : radiative limit to V_{OC}, measured using EQE_{EL}.

[c] ΔE_2 ((qV_{oc}^{SQ}-qV_{oc}^{rad}): voltage losses due to non-ideal absorption (it was calculated from EL and FTPS measurements).

[d] ΔE_3 ($q\Delta V_{oc}^{non-rad}$): voltage losses due to non-radiative recombination only.

Supplementary Table 5. Photovoltaic parameters of the flexible devices based on the three all-PSCs under the AM 1.5 G illumination of 100 mW cm^{-2}.

Material Combination	V_{OC} [V]	J_{SC} [mA cm^{-2}]	FF [%]	PCE [%]
PM6: PY-V-*γ*:	0.856	24.0	65.7	13.5
PM6: PY-V-*γ*:PCBM	0.865	24.3	66.6	14.0
PM6: PY-V-*γ*:PFBO-C12	0.870	24.6	68.2	14.6

Supplementary Table 6. Photovoltaic parameters of the rigid devices based on the PM6:PYF-T-*o*, PM6:PYF-T-*o*:PCBM and PM6:PYF-T-*o*:PFBO-C12 under the AM 1.5 G illumination of 100 mW cm^{-2}.

Material Combination	V_{OC} [V]	J_{SC} [mA cm^{-2}]	FF [%]	PCE [%]
PM6:PYF-T-*o*	0.915	24.8	71.5	16.2
PM6:PYF-T-*o*:PCBM	0.918	25.1	72.5	16.7
PM6:PYF-T-*o*:PFBO-C12	0.923	25.3	73.3	17.1

Supplementary Table 7. Photovoltaic parameters of the all-PSCs based on the PM6:PY-V-*γ*:PFBO-C12 with different weight ratio of PFBO-C12.

Weight ratio (%)	V_{OC} [V]	J_{SC} [mA cm^{-2}]	FF [%]	PCE [%]
5	0.902	25.3	76.3	17.4
10	0.905	25.8	77.0	18.0
20	0.906	25.6	76.8	17.8

3. Supplementary Methods

Synthesis of Compound 1

Hydroquinone (10 mmol, 1.1 g) and K_2CO_3 (30 mmol, 4.1 g) were added to acetonitrile (50 mL) in a single-necked flask and heated to reflux. After thirty minutes, the suspension became dark yellow, at which point bromododecane (30 mmol, 7.5 g) was added in one shot. It was then refluxed for 16 h under air. The mother liquor was poured into cold water, and the product recovered by filtration. Recrystallized from chloroform and methanol purified the product. After drying under reduced pressure, a white solid was recovered with a yield of 85%. ^{1}H NMR (400 MHz, CDCl3) δ (ppm): 6.82 (s, 4H), 3.88 – 3.91 (t, 4H), 1.72 – 1.79 (quint, 4H), 1.42 – 1.46 (m, 4H), 1.27 (s, 32H), 0.87-0.90 (t, 6H). ^{13}C NMR (101 MHz, CDCl3) δ (ppm): 153.22 (s), 115.41 (s), 68.69 (s), 31.94 (s), 29.65 (s), 29.62 (s), 29.61 (s), 29.44 (s), 29.43 (s), 29.37 (s), 26.08 (s), 22.71 (s), 14.13 (s). HRMS m/z: $[M]^+$ found 447.13367.

Synthesis of Compound 2

Paraformaldehyde (27.0 mmol, 0.8 g) and **1** (4.5 mmol, 2.0 g) and were added to acetic acid (50 mL) in a single-necked flask and heated to 80 ˚C. After dissolution, HBr (33 %, 4 mL) in acetic acid was added in one shot. The reactor was sealed and heated for 8 h. The reaction was then allowed to cool to room temperature; the precipitate was filtered
off and washed with water and methanol. The resulting solid was purified by recrystallization from acetonitrile, yielding 1.7 g (2.79 mmol, 62%) white solid. ^{1}H NMR (400 MHz, CDCl3) δ (ppm): 6.85 (s, 2H), 4.53 (s, 4H), 3.97-4.00 (t, 4H), 1.83 – 1.79 (quint, 4H), 1.51 – 1.46 (m, 4H), 1.36-1.27 (m, 32H), 0.87-0.90 (t, 6H). ^{13}C NMR (101 MHz, CDCl3) δ (ppm): 150.69 (s), 127.54 (s), 114.68 (s), 69.04 (s), 31.94 (s), 29.69 (s), 29.66 (s), 29.62 (s), 29.37 (s), 29.35 (s), 28.75 (s), 26.10 (s), 22.71 (s), 14.14 (s). HRMS m/z: $[M]^+$ found 632.44165.

Synthesis of PFBO-C12

The PFBO-C12 polymer was synthesized with an atom transfer radical addition polymerization reaction.[1] Into a flame-dried, N_2-flushed flask, 200 mL flask was placed C_{60} (120 mg, 0.167 mmol), freshly distilled toluene (72 mL), CuBr (0.334 mmol) and 2,2'-bipyridine (0.668 mmol) were stirred

at room temperature for 2 h. On confirmation of the solvation of C_{60}, the compound **2** (105 mg, 0.167 mmol) was added and the mixture quickly brought to reflux. Following stirring for 24 h at reflux and under cover from light, the toluene was removed under rotary evaporation. The solid deposit was dissolved in a minimum of 1,2-dichlorobenzene, precipitated by addition to methanol, and recovered by filtration in a cellulose tube ready for Soxhlet extraction with acetone (2 d) and n-hexane (2 d). The polymer was dried under reduced pressure to a brown powder (yield 60%). ^{1}H NMR (400 MHz, CDCl3) δ (ppm): 7.23 (aromatic), 7.18 (aromatic), 4.50 (Ar-CH_2), 3.83 (m, O-CH_2), 1.83 (Alkyl Chain's H), 1.16 (Alkyl Chain's H), 0.79 (CH_3).

4. Supplementary References

1. Silva, H. S. *et al.* Oligo- and poly(fullerene)s for photovoltaic applications: Modeled electronic behaviors and synthesis. *J. Polym. Sci., Part A: Polym. Chem.* **55**, 1345–1355 (2017).
2. Jia, T. *et al.* 14.4% efficiency all-polymer solar cell with broad absorption and low energy loss enabled by a novel polymer acceptor. *Nano Energy* **72**, 104718 (2020).
3. Wang, W. *et al.* Controlling molecular mass of low-band-gap polymer acceptors for high-performance all-polymer solar cells. *Joule* **4**, 1070-1086 (2020).
4. Fan, Q. *et al.* 10.13% Efficiency all-polymer solar cells enabled by improving the optical absorption of polymer acceptors. *Solar RRL* **4**, 2000142 (2020).
5. Du, J. *et al.* High-performance all-polymer solar cells: synthesis of polymer acceptor by a random ternary copolymerization strategy. *Angew. Chem. Int. Ed.* **59**, 15181-15185 (2020).
6. Sun, H. *et al.* A narrow-bandgap n-type polymer with an acceptor–acceptor backbone enabling efficient all-polymer solar cells. *Adv. Mater*. **32**, 2004183 (2020).
7. Fan, Q. *et al.* Over 14% efficiency all-polymer solar cells enabled by a low bandgap polymer acceptor with low energy loss and efficient charge separation. *Energy Environ. Sci.* **13**, 5017-5027 (2020).
8. Luo, Z. *et al.* Precisely controlling the position of bromine on the end group enables well-regular polymer acceptors for all-polymer solar cells with efficiencies over 15%. *Adv. Mater.* **32**, 2005942 (2020).

9. Peng, F. *et al.* A universal fluorinated polymer acceptor enables all-polymer solar cells with >15% efficiency. *ACS Energy Lett.* **5**, 3702 (2020).
10. Yu, H. *et al.* Fluorinated end group enables high-performance all-polymer solar cells with near-infrared absorption and enhanced device efficiency over 14%. *Adv. Energy Mater.* **11**, 2003171 (2021).
11. Yu, H. *et al.* Regio-regular polymer acceptors enabled by determined fluorination on end groups for all-polymer solar cells with 15.2 % efficiency. *Angew. Chem. Int. Ed.* **60**, 10137-10146 (2021).
12. Zhang, L. *et al.* 15.4% Efficiency all-polymer solar cells. *Sci. China Chem.* **64**, 408-412 (2021).
13. Wang, T. *et al.* Highly efficient and stable all-polymer solar cells enabled by near-infrared isomerized polymer acceptors. *Chem. Mater*. **33**, 761-773 (2021).
14. Su, N. *et al.* High-efficiency all-polymer solar cells with poly-small-molecule acceptors having π-extended units with broad near-IR absorption. *ACS Energy Lett.* **6**, 728-738 (2021).
15. Fu, H. *et al.* High efficiency (15.8%) all-polymer solar cells enabled by a regioregular narrow bandgap polymer acceptor. *J. Am. Chem. Soc.* **143**, 2665–2670 (2021).
16. Jia, T. *et al.* All-polymer solar cells with efficiency approaching 16% enabled using a dithieno[3′,2′:3,4;2″,3″:5,6]benzo[1,2-c][1,2,5]thiadiazole (fDTBT)-based polymer donor. *J. Mater. Chem. A* **9**, 8975-8983 (2021).
17. Liu, T. *et al.* 16% efficiency all-polymer organic solar cells enabled by a finely tuned morphology via the design of ternary blend. *Joule* **5**, 914–930 (2021).
18. Yu, H. *et al.* A difluoro-monobromo end group enables high-performance polymer acceptor and efficient all-polymer solar cells processable with green solvent under ambient condition. *Adv. Funct. Mater.* **31**, 2100791 (2021).
19. Du, J. *et al.* Polymerized small molecular acceptor based all-polymer solar cells with an efficiency of 16.16% via tuning polymer blend morphology by molecular design. *Nat. Comm.* **12**, 5264 (2021).

20. Zhang J. *et al.* π-extended conjugated polymer acceptor containing thienylene–vinylene–thienylene unit for high-performance thick-film all-polymer solar cells with superior long-term stability. *Adv. Energy Mater.* **11**, 2102559 (2021).

21. Wu, Q. *et al.* Tailoring polymer acceptors by electron linkers for achieving efficient and stable all-polymer solar cells. *Natl. Sci. Rev.* **9**, nwab151 (2022).

22. Fan, Q. *et al.* Multi-selenophene-containing narrow bandgap polymer acceptors for all-polymer solar cells with over 15 % efficiency and high reproducibility. *Angew. Chem. Int. Ed.* **60**, 15935-15943 (2021).

23. Fan, Q. *et al.* Near-infrared absorbing polymer acceptors enabled by selenophene-fused core and halogenated end-group for binary all-polymer solar cells with efficiency over 16%. *Nano Energy* **92**, 106718 (2021).

24. Fu, H. *et al.* 16.3% Efficiency binary all-polymer solar cells enabled by a novel polymer acceptor with an asymmetrical selenophene-fused backbone. *Sci. China Chem.* **65**, 309-317 (2021).

25. Chen, D. *et al.* Printable and stable all-polymer solar cells based on non-conjugated polymer acceptors with excellent mechanical robustness. *Sci. China Chem.* **65**, 182-189 (2022).

26. Zhou, L. *et al.* Introducing electron-withdrawing linking units and thiophene π-bridges into polymerized small molecule acceptors for high-efficiency all-polymer solar cells. *Chem. Mater.* **33**, 8212-8222 (2021).

27. Seo, S. *et al.* Importance of high-electron mobility in polymer acceptors for efficient all-polymer solar cells: combined engineering of backbone building unit and regioregularity. *Adv. Funct. Mater.* **32**, 2108508 (2022).

28. Li, Y. *et al.* Polymerized small molecular acceptor with branched side chains for all polymer solar cells with efficiency over 16.7%. *Adv. Mater.* **34**, 2110155 (2022).

29. Jia, J. *et al.* Fine-tuning batch factors of polymer acceptors enables a binary all-polymer solar cell with high efficiency of 16.11%. *Adv. Energy Mater.* **12**, 2103193 (2022).

30. Genene, Z. *et al.* Polymer acceptors with flexible spacers afford efficient and mechanically robust all-polymer solar cells. *Adv. Mater.* **34**, 2107361 (2022).
