# Supplementary material for: Improved photovoltaic performance and robustness of all-polymer solar cells enabled by a polyfullerene guest acceptor
Source: Nat Commun. 2023 Apr 22;14:2323. doi: 10.1038/s41467-023-37738-9 (PMC10122667; doi:10.1038/s41467-023-37738-9)
Supplement: Supplementary file 2 — Reporting Summary [file 41467_2023_37738_MOESM2_ESM.pdf]

nature research

Corresponding author(s): He Yan

Solar Cells Reporting Summary

Nature Research wishes to improve the reproducibility of the work that we publish. This form is intended for publication with all accepted papers reporting the characterization of photovoltaic devices and provides structure for consistency and transparency in reporting. Some list items might not apply to an individual manuscript, but all fields must be completed for clarity.

For further information on Nature Research policies, including our data availability policy, see Authors & Referees.

Experimental design

Please check: are the following details reported in the manuscript?

1. Dimensions

Item	Yes/No	Response
Area of the tested solar cells	☒ Yes ☐ No	The area of the devices is 0.0644 cm^2.
Method used to determine the device area	☒ Yes ☐ No	A shadow mask is used to refine the device area to be 0.04 cm^2.

2. Current-voltage characterization

Item	Yes/No	Response
Current density-voltage (J-V) plots in both forward and backward direction	☐ Yes ☒ No	The OPV devices do not need both direction scan.
Voltage scan conditions<br>*For instance: scan direction, speed, dwell times*	☒ Yes ☐ No	forward, 0.01 V each step, 10 ms
Test environment<br>*For instance: characterization temperature, in air or in glove box*	☒ Yes ☐ No	room temperature, in the glove box
Protocol for preconditioning of the device before its characterization	☐ Yes ☒ No	*Explain why this information is not reported/not relevant.*
Stability of the J-V characteristic<br>*Verified with time evolution of the maximum power point or with the photocurrent at maximum power point; see ref. 7 for details.*	☐ Yes ☒ No	*Explain why this information is not reported/not relevant.*

3. Hysteresis or any other unusual behaviour

Item	Yes/No	Response
Description of the unusual behaviour observed during the characterization	☐ Yes ☒ No	*Explain why this information is not reported/not relevant.*
Related experimental data	☐ Yes ☒ No	*Explain why this information is not reported/not relevant.*

4. Efficiency

Item	Yes/No	Response
External quantum efficiency (EQE) or incident photons to current efficiency (IPCE)	☒ Yes ☐ No	The EQE is shown in Figure 2c.
A comparison between the integrated response under the standard reference spectrum and the response measure under the simulator	☒ Yes ☐ No	Within 3% variance
For tandem solar cells, the bias illumination and bias voltage used for each subcell	☐ Yes ☒ No	*Explain why this information is not reported/not relevant.*

5. Calibration

Item	Yes/No	Response
Light source and reference cell or sensor used for the characterization	☒ Yes ☐ No	The standard silicon solar cell is applied.
Confirmation that the reference cell was calibrated and certified	☒ Yes ☐ No	The standard current value is 75.64 mA.

Calculation of spectral mismatch between the reference cell and the devices under test

☐ Yes ☒ No

Explain why this information is not reported/not relevant.

6. Mask/aperture

Size of the mask/aperture used during testing

☒ Yes ☐ No

0.04 cm^2

Variation of the measured short-circuit current density with the mask/aperture area

☒ Yes ☐ No

within 2% varience

7. Performance certification

Identity of the independent certification laboratory that confirmed the photovoltaic performance

☒ Yes ☐ No

The devices have been fabricated in HKUST and CityU

A copy of any certificate(s)
Provide in Supplementary Information

☐ Yes ☒ No

Explain why this information is not reported/not relevant.

8. Statistics

Number of solar cells tested

☒ Yes ☐ No

15 devices

Statistical analysis of the device performance

☒ Yes ☐ No

State where this information can be found in the text.

9. Long-term stability analysis

Type of analysis, bias conditions and environmental conditions
For instance: illumination type, temperature, atmosphere humidity, encapsulation method, preconditioning temperature

☒ Yes ☐ No

Thermal Stability, mechanical stabilities
